# Fermentation couples *Chloroflexi* and sulfate-reducing bacteria to *Cyanobacteria* in hypersaline microbial mats

**DOI:** 10.3389/fmicb.2014.00061

**Published:** 2014-02-26

**Authors:** Jackson Z. Lee, Luke C. Burow, Dagmar Woebken, R. Craig Everroad, Mike D. Kubo, Alfred M. Spormann, Peter K. Weber, Jennifer Pett-Ridge, Brad M. Bebout, Tori M. Hoehler

**Affiliations:** ^1^Exobiology Branch, NASA Ames Research CenterMoffett Field, CA, USA; ^2^Bay Area Environmental Research InstituteSonoma, CA, USA; ^3^Departments of Civil and Environmental Engineering, and Chemical Engineering, Stanford UniversityStanford, CA, USA; ^4^The SETI InstituteMountain View, CA, USA; ^5^Lawrence Livermore National Lab, Chemical Sciences DivisionLivermore, CA, USA

**Keywords:** microbial mats, hydrogen, fermentation, Guerrero Negro, NanoSIMS

## Abstract

Past studies of hydrogen cycling in hypersaline microbial mats have shown an active nighttime cycle, with production largely from *Cyanobacteria* and consumption from sulfate-reducing bacteria (SRB). However, the mechanisms and magnitude of hydrogen cycling have not been extensively studied. Two mats types near Guerrero Negro, Mexico—permanently submerged *Microcoleus* microbial mat (GN-S), and intertidal *Lyngbya* microbial mat (GN-I)—were used in microcosm diel manipulation experiments with 3-(3,4-dichlorophenyl)-1,1-dimethylurea (DCMU), molybdate, ammonium addition, and physical disruption to understand the processes responsible for hydrogen cycling between mat microbes. Across microcosms, H_2_ production occurred under dark anoxic conditions with simultaneous production of a suite of organic acids. H_2_ production was not significantly affected by inhibition of nitrogen fixation, but rather appears to result from constitutive fermentation of photosynthetic storage products by oxygenic phototrophs. Comparison to accumulated glycogen and to CO_2_ flux indicated that, in the GN-I mat, fermentation released almost all of the carbon fixed via photosynthesis during the preceding day, primarily as organic acids. Across mats, although oxygenic and anoxygenic phototrophs were detected, cyanobacterial [NiFe]-hydrogenase transcripts predominated. Molybdate inhibition experiments indicated that SRBs from a wide distribution of DsrA phylotypes were responsible for H_2_ consumption. Incubation with ^13^C-acetate and NanoSIMS (secondary ion mass-spectrometry) indicated higher uptake in both *Chloroflexi* and SRBs relative to other filamentous bacteria. These manipulations and diel incubations confirm that *Cyanobacteria* were the main fermenters in Guerrero Negro mats and that the net flux of nighttime fermentation byproducts (not only hydrogen) was largely regulated by the interplay between *Cyanobacteria*, SRBs, and *Chloroflexi*.

## Introduction

Hypersaline microbial mats, living analogs of early life on Earth (Des Marais, 2003), are compact and structured laminations of highly diverse microbial communities that undergo significant redox changes over the diel (day-night) cycle, alternating between oxic and anoxic states. The metabolic diversity of microbial mats is reflected in a diverse potential for H_2_ metabolism (Hoehler, 2005), and nitrogen fixation (Omoregie et al., 2004a,b), and previous work has documented significant efflux of H_2_ from hypersaline microbial mats (Hoehler et al., 2001; Burow et al., 2012) under dark anoxic conditions. Hence, these systems are of interest not only in an ecological frame of reference, but also for bioenergy science. In previous work we showed that nighttime production of hydrogen gas from hypersaline mats of Elkhorn Slough, CA, USA originated within the photic layer of the mats and was primarily attributable to the fermentation activity of *Cyanobacteria*, especially the dominant filamentous cyanobacterium *Microcoleus chthonoplastes*, and was largely insensitive to nitrogen fixation (Burow et al., 2012). In the Elkhorn Slough mats, hydrogen consumers [sulfate-reducing bacteria (SRB)] were present in close physical association with hydrogen producers, and significantly reduced hydrogen efflux (Burow et al., 2013, in press). Understanding the ecological and environmental factors that control net H_2_ production is thus critical to understanding the role of H_2_ cycling in mat structure and ecology.

In the present work we have characterized and quantified fermentative activity and consumption of fermentation products in two mat types from the previously documented site at Guerrero Negro, B.C.S., Mexico, that exhibit a range of net H_2_ production rates. “GN-S” are well-developed subtidal mats located in pond 4 near pond 5 of the salt works and constructed primarily by the cyanobacterium *Microcoleus chthonoplastes* that have been described extensively in previous reports (Spear et al., 2003; Ley et al., 2006; Feazel et al., 2008; and Robertson et al., 2009). “GN-I” are intertidal mat communities constructed largely by *Lyngbya* sp. The difference in dominant cyanobacterium and extent of development of accessory populations are reflected in differing chemical behavior of the two mat types, including in H_2_ efflux. Previous work on the GN-I mats documented that the integrated H_2_ production rate is equivalent to 16% of net daytime carbon fixation (on a per-electron basis), and individual bubbles at the mat surface may contain up to 10% hydrogen in the pre-dawn hours (Hoehler et al., 2001; Hoehler, 2005). Moreover, the amount of H_2_ efflux has been found to vary over more than four orders of magnitude, as a function of environmental forcing (Bebout et al., 2002, 2004; Hoehler, 2005; Burow et al., 2012). In comparison, the submerged mats experience relatively stable conditions and release less H_2_.

The extensive diversity, dominance patterns, and dynamic microbial response over space and time found within microbial mats are well suited for the development and deployment of advanced sequencing and isotope probing techniques to identify the complex biological interactions as well as energy and nutrient cycles of these highly diverse systems. When combined with traditional biogeochemical techniques, these methods can provide insights into energy and nutrient cycling in and through these systems. Using a comparative approach, the relative role of fermentation in the carbon and hydrogen cycles of these two mat types, and the ecology surrounding these cycles, was examined via a holistic set of molecular, isotopic, and biogeochemical methods. Specifically, we employed pyrotag libraries, functional gene sequencing of [NiFe]-hydrogenase (*hoxH*) and dissimilatory sulfite reductase (*dsrA*), stable isotope probing of labeled ^13^C-bicarbonate and ^13^C-acetate, Catalyzed Reporter Deposition Fluorescence In Situ Hybridization (CARD-FISH) probing of *Chloroflexi* and SRB clades, in combination with measurements of hydrogen, hydrogen sulfide, and organic acids to examine microbial mats manipulated with inhibitors that disrupt the sulfur, nitrogen, and carbon cycles. The results from these experiments indicated that in GN-I mats constitutive fermentation served to liberate roughly 80% of the photosynthetically fixed electrons into the bulk pool and thereby formed a basis for close trophic coupling between *Cyanobacteria*, filamentous anoxygenic phototrophs, and SRBs in hypersaline mats of both Guerrero Negro and of Elkhorn Slough.

## Materials and methods

### Field site and sample collection

Water and whole 30 × 30 cm sections of microbial mats were harvested in September 2010 and September 2011 from sites located near Guerrero Negro, B.C.S. Mexico. Two distinct mat types were selected for study. Mats from the seawater concentration area (pond 4 near 5) of the Exportadora de Sal, S.A. (ESSA) Guerrero Negro, B.C.S. saltworks (27° 41′ 20.6″ N, 113° 55′ 1.2′ W) have a well-documented community profile (Spear et al., 2003; Ley et al., 2006; Feazel et al., 2008; and Robertson et al., 2009). These mats have experienced a largely quiescent environment with permanent cover of approximately 0.5–1 m of water at ~80–100‰ salinity, and can grow to approximately 10 cm thick (Des Marais, 1995; Nübel et al., 1999). This mat type, which is constructed primarily by the cyanobacterium *Microcoleus chthonoplastes* is referred in this study as “GN-S” (Guerrero Negro—Submerged). The second mat type was collected from the intertidal flats bordering Laguna Ojo de Liebre, just outside the ESSA salt works (27° 45′ 30.2″ N, 113° 59′ 42.8″ W), and is referred to as “GN-I” mats (Guerrero Negro—Intertidal). The GN-I mats, constructed primarily by the cyanobacterium *Lyngbya* spp., experience periodic tidal desiccation and breakup along a sandy sloping shore ecological gradient (Rothrock and Garcia-Pichel, 2005). Mats were also collected from Elkhorn Slough, CA, USA in November 2011 in order to facilitate comparison of the present work with previously published studies of microbial mat fermentation and H_2_ production and consumption (Burow et al., 2012; Woebken et al., 2012). Samples were returned to a greenhouse facility at NASA Ames and maintained in UV transparent acrylic boxes under ~3 cm of water collected from the site specific to the individual mat types as previously described (Bebout et al., 2002). The mats received natural solar irradiance and regulated temperature environment designed to mimic natural daily fluctuations around the *in situ* average of ~19°C.

### Diel manipulations

Diel (24 hour) studies of these mat types were performed with inhibitors of specific metabolic processes and in conjunction with physical disruption by homogenization, all conducted under natural light conditions. All manipulations were performed by placing replicate 11 mm diameter cores of the top 2 mm of the microbial mats into 14 ml glass serum vials with 4 ml site water. Bottles were closed with butyl rubber stoppers, crimp sealed, flushed with nitrogen gas, and incubated under controlled temperature (Burow et al., 2012). Six replicates were prepared for each experimental condition. The four distinct manipulation experiments were:

1. DCMU [3-(3,4-dichlorophenyl)-1,1-dimethylurea, a specific inhibitor of Photosystem II (Oremland and Capone, 1988; Oettmeier, 1992), and therefore of oxygenic photosynthesis] was used to evaluate the contribution of oxygenic photosynthetic carbon fixation as a driver of the release of fermentation products. DCMU was added to mats at dawn at a final concentration of 20 μM.
2. Ammonium chloride was added as a source of combined nitrogen in order to suppress dinitrogen-fixing activity (Bebout et al., 1987; Burow et al., 2012) and thereby investigate whether N-fixation activity (and associated energy demand) affected rates of fermentation. Ammonium chloride was added at dusk at a final concentration of 8.8 mM.
3. Sodium molybdate, a competitive inhibitor of sulfate reduction (Oremland and Capone, 1988), was used to quantify the role of sulfate reducers in consuming fermentation products. Sodium molybdate was added to mat incubations in site water and in sulfate-free artificial ASN5 media (to enhance the competitive effect) at dusk at a final concentration of 30 mM.
4. Mat microbes were physically disrupted from each other with a Tissue Master (TM125-115, Omni International, Kennesaw, GA, USA) in order to investigate the importance of close spatial associations between microbes in internal cycling vs. diffusive loss of fermentation products (Burow et al., in press).

### Biogeochemical methods

#### Analysis of H_2_ and organic acids

To measure H_2_ net flux, 25 μ L of headspace was withdrawn with a volumetric syringe from the headspace of each replicate microcosm vial at several time points during an incubation period. Samples were either analyzed immediately by direct injection onto a gas chromatograph with an HgO reduction detector (Trace Analytical) (Burow et al., 2012), or were preserved for later analysis as a small (1 mL) gas sample in a serum vial containing saturated NaCl solution that had been sparged with nitrogen gas for 20 min. To analyze organic acids, the entire liquid phase (4 mL) of each of three replicate microcosms for each control or manipulation experiment was sampled (with the associated incubation sacrificed). Liquid was filtered through 0.2 μm syringe filters for storage in ashed glass vials at −20°C. Organic acids (C1–C5) were quantified via high-pressure liquid chromatography (Albert and Martens, 1997).

#### Analysis of glycogen, dissolved inorganic carbon (DIC), and hydrogen sulfide

Whole 1-cm diameter subcores were collected from incubated microbial mats and immediately frozen in liquid nitrogen. The uppermost 2 mm was sub-sectioned while still frozen and was subsequently freeze dried. The dried samples were ground and sonicated (3 min, setting 6, Fisher 60 Dismembrator) and transferred to a 7 ml glass screwcap vial. The vial was then capped and put in a boiling water bath for 6 min to solubilize glycogen and stop any enzyme activity in the extract. 50 μl of the extract was filter sterilized (0.45 μm) and added to 50 μl of amyloglucosidase solution (Keppler and Decker, 1974) (0.01 g in 2 ml deionized water) in a 1.5 ml screwcap vial. The vial was placed horizontally in a 40°C heating block on an orbital shaker so that the vial could roll slightly to aid mixing. After 1 h, the vial received 100 μl of a derivatization solution consisting of 60 mg anthranilamide (Sigma-Aldrich, St. Louis, MO, USA), 40 mg sodium cyanoborohydride (Sigma-Aldrich), 0.6 ml glacial acetic acid and 1.4 ml dimethyl sulfoxide (Bigge et al., 1995) and then heated to 70°C for 1 h followed by isocratic HPLC separation and fluorescence detection (270 nm excitation, 430 nm emission). The solvent consisted of 20 ml tetrahydrofuran, 6 ml butylamine, 10 ml phosphoric acid, 12 ml tetraethyl ammonium hydroxide and 3950 ml deionized water (Anumula, 1994).

DIC was quantified in separate flux chamber experiments of whole mats based on the methods of Hoehler et al. (2001). 1.5 mL fluid samples were collected in 3 mL plastic syringes from 1.5 L flux chambers placed on mats in the greenhouse, closed by means of a 3-way stopcock, and stored at 4°C until analysis (typically within 2–3 h, but in no case greater than 48 h after collection). Samples were analyzed via flow injection analyzer (FIA) (Hall and Aller, 1992). Duplicate injections were made for each incubation time point but, due to limited volume of incubation fluids, replicate samples were not taken.

Hydrogen sulfide measurements were based on the work of Cline (1969) and consisted of a *N,N*-Dimethyl-*p*-phenylenediamine sulfate salt and iron(III) chloride colorimetric method (read at 670 nm) with a sodium sulfide standard.

### Photosynthesis/fermentation mass and electron balance

Net flux data collected from several diel experiments were used to estimate the proportion of photosynthetic carbon uptake that was subsequently mobilized in fermentation. We compared DIC uptake and flux of fermentation products on the common basis of “electron equivalents” to enable us to include H_2_ in the calculation. The flux amounts were used to determine total daytime and nighttime DIC flux and averaged to obtain the average flux of inorganic carbon taken up and released by mats over a single diel cycle. Glycogen day and night differences were averaged and used to estimate net fixed carbon accumulation and depletion. Net and total production of organic acids and hydrogen were estimated, respectively, from replicated control and physical disruption experiments. All fluxes were normalized to the surface area of incubated mat cores and then converted to “electron equivalents” based on total charge state [(CH_2_O)_n_ = 4n, CH_3_COOH = 8, etc.] of each chemical species. Photoautotrophy was assumed to fix four electrons per carbon. Standard deviations were distributions of replicate measurements propagated to final results.

### Molecular biology methods

#### Nucleic acid isolation and pyrotag sequencing of 16s small subunit (SSU) rRNA genes and transcripts

In October 2011 (1 month after field collection), samples for pyrotag libraries of both GN mat types were collected at 1200–2400 h from unaltered mats kept in site water in a greenhouse environment. Nucleic acid extraction [RNA clean extractions for nucleic acids (DNA and RNA) followed by cDNA reverse-transcription] was performed as per Burow et al. (2012). The upper 2 mm phototrophic layer was homogenized and extracted using acid-phenol and DNA/RNA cleanup and separation with the Qiagen RNeasy Mini Kit and the QIAamp DNA Mini Kit as per the manufacturers' protocol (Qiagen, Venlo, The Netherlands). Extractions were done in triplicate and pooled. The V8 hypervariable region of the 16S SSU rRNA gene was amplified from DNA (rRNA gene) or cDNA (rRNA reverse-transcript) templates using the universal primer pair 926f/1392r (Engelbrektson et al., 2010), including the titanium adaptor sequences and a five-base barcode on the reverse primer. Sequencing and bioinformatics processing was completed by Research and Testing Laboratory, LLC (Lubbock, TX, USA) using an in-house denoising, demultiplexing, clustering, and taxonomic assignment pipeline (http://www.researchandtesting.com/). Operational Taxonomic Unit (OTU) tables of denoised and dereplicated sequences with taxonomies identified by BLASTN+ on a custom NCBI database were received from the vendor. Taxonomy and taxonomic levels were based on BLAST percent similarity to best match reference sequences (same species = 97% ID, genus = 95%, etc.). Population statistics were computed using subsampling (*n* = 100) to the smallest library size (11,500) using QIIME (Caporaso et al., 2010) and the average and distribution of Chao1 and ACE metrics determined. Interactive Krona HTML5 (Ondov et al., 2011) hierarchical pie chart community profiles of all pyrotag libraries have been included in the supplemental information online as Krona_charts_supplemental.zip.

#### Degenerate bidirectional [NiFe]-hydrogenase (HOXH) and dissimilatory sulfite reductase (dsrA) gene sequencing

Primers HoxH_F37 (5′ -ATHGARGGHCAYGCBAARAT-3′) and HoxH_R518 (5′ -ACNCCICCVGGNAYHGHCCA-3′) developed to amplify reversible nickel-iron (type 3b) hydrogenase transcripts containing the L1 and L2 motifs (Vignais and Billoud, 2007) and used previously (Burow et al., 2012) were used to sequence hydrogenases from both mat types from nighttime manipulations (2 96-well plates each from GN-I and GN-S). Primers dsrA_1R (5′-ACSCACTGGAAGCACG-3′) (Wagner et al., 1998) and dsrA_DGGE_R (5′- CGGTGMAGYTCRTC −3′) (Leloup et al., 2009) designed to detect sulfate reduction capability from SRBs were used to sequence *dsrA* genes from both mat types in nighttime manipulations (Burow et al., in press) (2 cDNA and 1 DNA 96-well plates each from GN-I and GN-S). PCR and cloning (described in Burow et al., 2012) were used to prepare 96-well format clone libraries of both DNA and cDNA for expression ratio profiling. Sequencing was completed using Single Pass T3 primer end sequencing runs at Beckman Coulter Genomics (Danvers, MA, USA) using BigDye Terminator v3.1 sequencing on an ABI PRISM 3730*xl* (Life Technologies, Carlsbad, CA, USA).

#### Phylogenetic analysis of bidirectional [nife]-hydrogenase (HOXH) and dissimilatory sulfite reductase (dsrA) clone sequences

All sequences were quality trimmed (Q30, 10-base average), and filtered for primer and plasmid regions and translated to the amino acid coding frame using Geneious (Biomatters, Auckland, New Zealand). Sequences were exported and then clustered by CD-HIT (Li and Godzik, 2006) at the 97% similarity level and queried against the NCBI non-redundant peptide database using the Basic Local Alignment Search Tool (BLAST) (Altschul et al., 1990). Sequences were then aligned to custom databases composed of reference *dsrA* or *hoxH* sequences from environmental samples and isolated bacteria using CLUSTALX2 (Larkin et al., 2007) and then manually curated. The final alignment, excluding gapped positions, included 134 residues for 40 OTUs and 84 reference sequences for *dsrA* and 156 residues for 17 OTUs and 46 reference sequences for *hoxH*. For *hoxH* genes, 4 high-variability amino acid positions in the alignment (<50% conserved) were removed from the alignment. For each gene, maximum likelihood phylogenetic trees were constructed using PhyML (Guindon et al., 2010) with the LG+I+G substitution matrix as selected using the AIC criterion using Prottest 2.4 (Abascal et al., 2005; Le and Gascuel, 2008). Neighbor-joining was performed in MEGA5.2 (Tamura et al., 2011) using the JTT+I+G substitution matrix (Jones et al., 1992), which was the best-fit matrix available, as determined by Prottest. ML and NJ analyses underwent 100 and 1000 bootstrap iterations, respectively. Deeply branching or closely related reference sequences were pruned from trees for readability.

### Data archiving

Representative clone sequences of *hoxH* and *dsrA* OTUs were submitted to GenBank as accession KF582421-KF582479. Pyrotag libraries were submitted as ^*^.sff files to NCBI's Sequence Read Archive (SRA) under BioProject PRJNA219681 (accession SRP030038).

### Isotope labeling methods

#### ^13^C-bicarbonate and ^13^C-acetate labeling of GN mats and isotope-ratio mass spectrometry (IRMS)

Small subcores (11-mm diameter, 2-mm depth) of each microbial mat type were cut from whole sections of intact microbial mat and placed in serum vials with 4 ml site water. ^13^C-bicarbonate was added for 10 daytime hours to examine autotrophic light-driven incorporation. [2-^13^C]-acetate (0.2 mM) labeled FISH-NanoSIMS was completed according to Burow et al. (2012) and consisted of 10 hour overnight incubations in microcosm vials. Unlabeled mat sections, paraformaldehyde (PFA) fixed mat cores, and continuously dark microcosms served as controls. Mat cores were washed twice in label free media and immediately flash frozen at −80°C prior to FISH and NanoSIMS analyses. Bulk isotope ratios for ^13^C/^12^C (reported as δ^13^C in permil relative to VPDB) were determined by IRMS (ANCA-IRMS; PDZ Europa Limited, Crewe, England) at the University of California, Berkeley, with IAEA and NIST peach leaf standards used for C isotope standard corrections (Woebken et al., 2012). For each mat type, IRMS measurements were analyzed for significant differences using Student's *t*-test between different trials.

#### FISH and NanoSIMS

As previously described, CARD-FISH was used to hybridize fluorescent oligonucleotides to cells of *Chloroflexi* (CFX1223 and GNSB-941) (Woebken et al., 2012; Burow et al., 2013) and *Desulfosarcina* / *Desulfobacteraceae* (DSS658) (Burow et al., in press). *M. chthonoplastes* bundles were directly identified by morphology using scanning electron microscopy (SEM) on a FEI Inspect F (Hillsboro, Oregon, USA). Using the FISH, Chlorophyll *a* autofluorescence, and SEM micrographs as a guide to the location of specific cells types, high-resolution secondary ion mass spectrometry (SIMS) was performed at Lawrence Livermore National Laboratory with a Cameca NanoSIMS 50 (Gennevilliers, France). NanoSIMS data were analyzed for normal distribution with the Student's *t*-test and Shapiro-Wilk W test, and in cases where the data did not meet the standard (*p* < 0.05) of normality, the Wilcoxon (Whitney-Mann) test was used to confirm the initial result of significant differences using Student's *t*-test. Statistical tests were computed in R (2.15.1) (Ihaka and Gentleman, 1996). The ^13^C/^12^C ratio was measured using ^12^C_2_ and ^13^C/^12^C corrected for the dimer abundances and converted to permil enrichment (Pett-Ridge and Weber, 2012). Measurements were made in both imaging and spot analysis modes. Uptake of the respective ^13^C-labeled compound was determined based on ^13^C enrichment relative to unlabeled, PFA fixed samples as a reference.

## Results

### Pyrotag assay results of GN-I and GN-S mats

**Figure 1:**
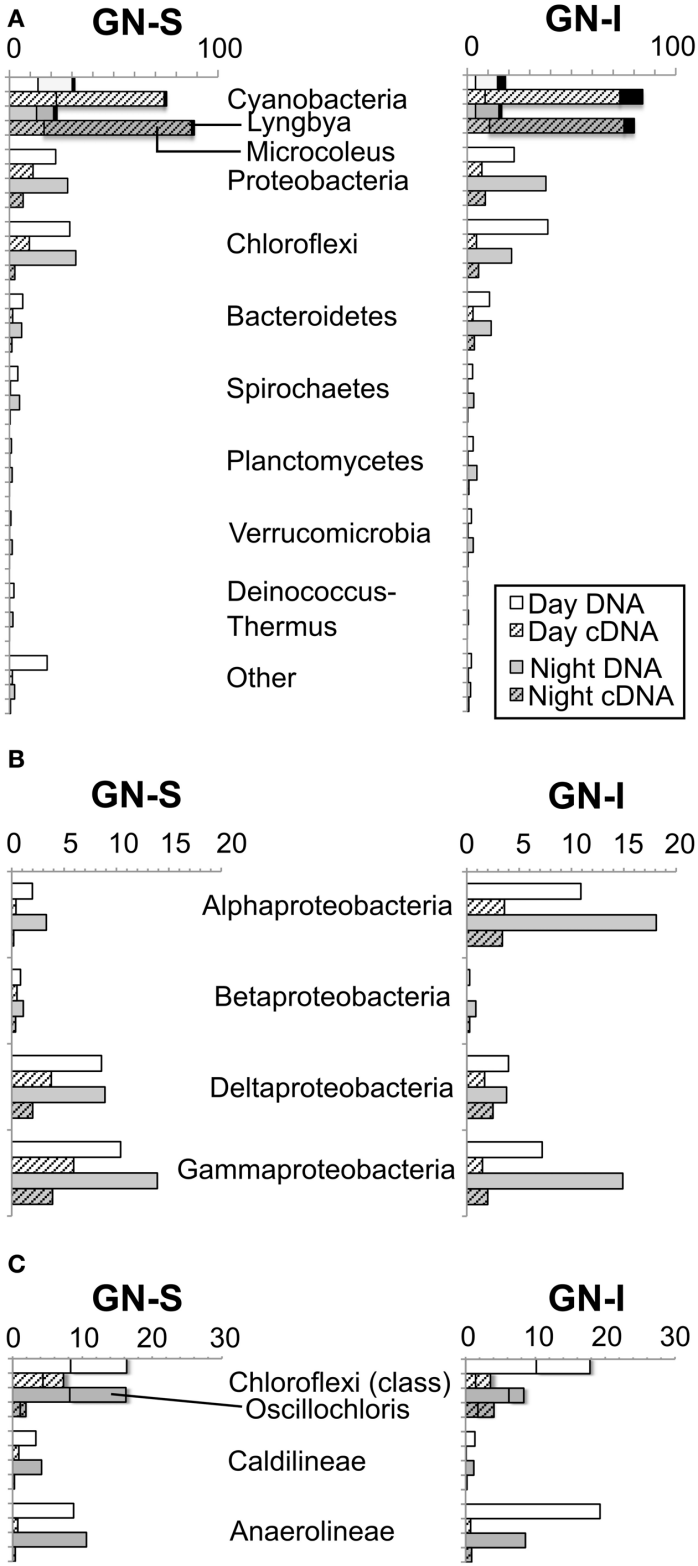
**Relative abundance of pyrotag sequences of intertidal (GN-I) and submerged (GN-S) microbial mats from Guerrero Negro, B.C.S., Mexico. (A)** Phylum level overview showing most abundant and most active OTUs [*Microcoleus* (shadowed), *Lyngbya* (black)]; **(B)** abundance within *Proteobacteria*; and **(C)** abundance within *Chloroflexi* with shadowing for *Oscillochloris*.

16S SSU rRNA gene pyrotag libraries provide a way to survey a large fraction of a microbial community to determine community composition and population statistics, as well as to track population changes between samples (Hamady et al., 2008; Kuczynski et al., 2010). Table 1 shows the estimated OTU richness and sequencing depth of the samples collected at 1200 and 2400 over the same diel cycle. DNA libraries were much more diverse than cDNA libraries and indicated that a small number of clades were responsible for a large fraction of the ribosomal expression in mats. Overall, coverage based on Chao1 and ACE estimators was approximately half in samples, though the proper usage of alpha diversity estimators in high-throughput sequencing studies (combined with denoising or clustering) is still debated (Reeder and Knight, 2010; Gihring et al., 2012) and therefore should be interpreted as a preliminary measure of coverage. Assuming that dominant organisms received adequate sequencing coverage, only OTU expression ratios (cDNA: DNA) of genera >1% in DNA libraries were examined during downstream analysis and only the top genera in phyla were highlighted. In Figure 1A, phylum level results of pyrotag libraries showed *Proteobacteria*, *Cyanobacteria*, and *Chloroflexi* dominate the DNA libraries, with relatively lower levels of *Bacteroidetes* than in previously sequenced GN-S samples (Ley et al., 2006). In GN-S mats, the OTUs from the genus *Microcoleus* had the highest expression ratio, and in GN-I mats, OTUs from the genus *Lyngbya* (followed by *Microcoleus*) had the highest expression ratio. While overall *Alphaproteobacteria* community composition was similar between the two GN mat types (Figure 1B), (primarily diazotrophic and purple non-sulfur (PNS) bacteria, Online Information, Krona pie charts), there were generally more *Alphaproteobacteria* sequences in GN-I samples than in GN-S samples. Additionally, *Proteobacteria* represented the largest fraction of the community, with major mat functional groups (Fenchel and Finlay, 1995) represented (i.e., PNS in *Alphaproteobacteria*, SRBs in *Deltaproteobacteria*, sulfur-oxidizing bacteria in *Gammaproteobacteria*) (Online Information). The third-most abundant phyla in the mats, *Chloroflexi* (Figure 1C), was divided between the phototrophic (class *Chloroflexi*) and the dark filamentous clades of the *Anaerolineae* and *Caldilineae*. The most abundant identifiable genus level OTUs from phylum *Chloroflexi* matched reference sequences for the filamentous anoxygenic phototrophic *Oscillochloris* (and the *Oscillochloridaceae* generally, Online Information).

**Table 1:** **OTU richness and sampling depth of pyrotag samples from GN-S and GN-I mats**.

**Library**	**OTU richness**		
	**Observed species**	**Chao1**	**ACE**
		**Avg. (*SD*)**	**Avg. (*SD*)**
GN-S 1200 DNA	3042	5252 (49)	5743 (41)
GN-S 1200 cDNA	1944	3818 (179)	4124 (154)
GN-S 2400 DNA	3326	6068 (137)	6766 (123)
GN-S 2400 cDNA	1243	2322 (121)	2511 (97)
GN-I 1200 DNA	3417	6747 (180)	7465 (189)
GN-I 1200 cDNA	1404	2511 (105)	2643 (88)
GN-I 2400 DNA	3559	6679 (124)	7285 (120)
GN-I 2400 cDNA	1545	2840 (128)	3016 (107)

*Average population statistics are shown with standard deviations of subsample distributions (n = 100, 11,500 sequences) in parentheses. Observed Species statistics were computed at the full sampling depth*.

### Microcosm studies of the impact of nitrogen fixation and oxygenic photosynthesis on fermentation

**Figure 2:**
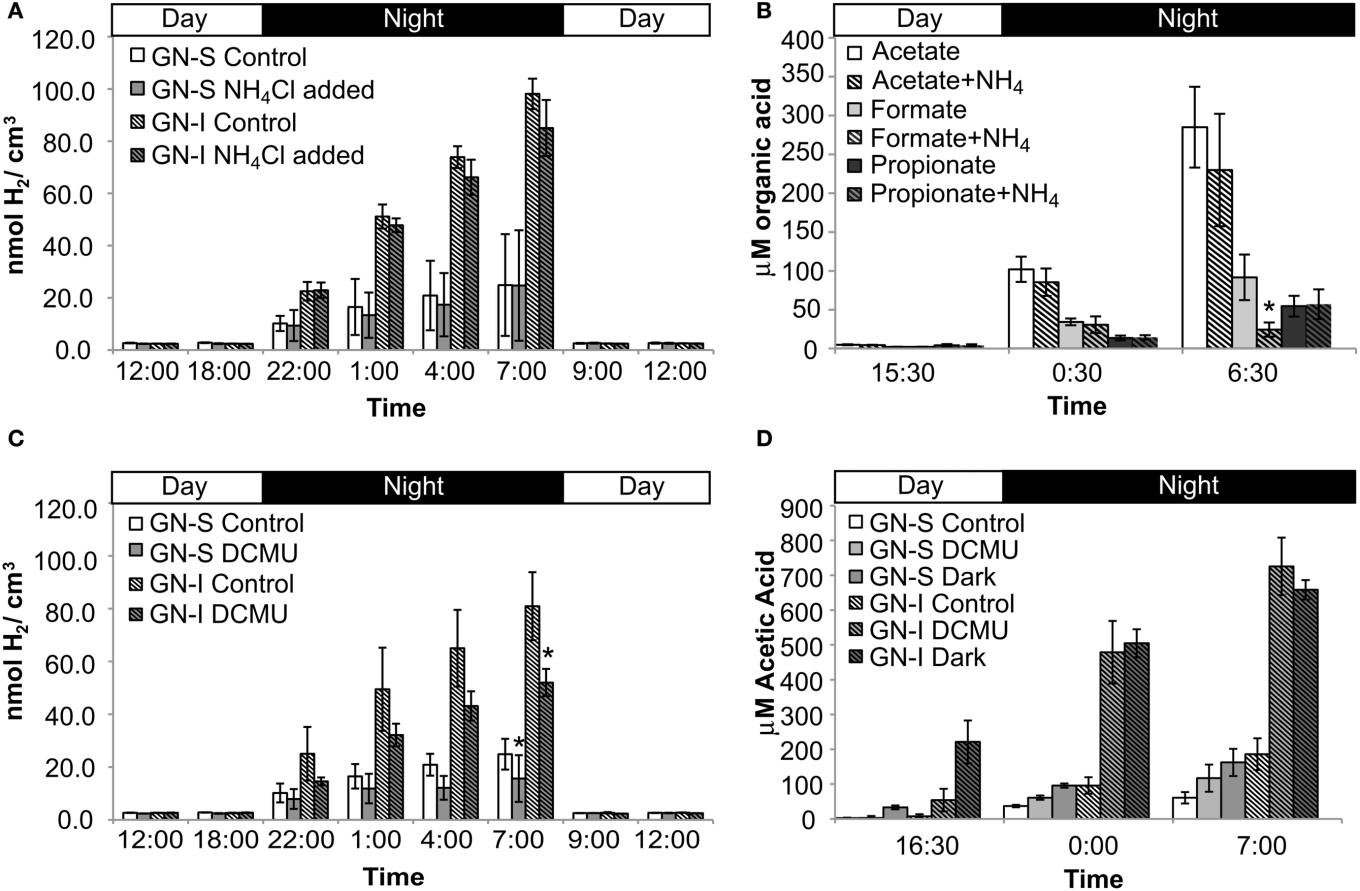
**Hydrogen and organic acid production in diel manipulation experiments with two microbial mats types from Guerrero Negro, B.C.S., Mexico**. NH_4_Cl manipulation (inhibition of nitrogen fixation): **(A)** H_2_ production **(B)** net organic acid production (GN-I only). DCMU manipulation (photosynthesis inhibition via photosystem II disruption): **(C)** H_2_ production and **(D)** net acetic acid production. Error bars indicate standard deviation of 6 replicate trials. Asterisk indicates *p* < 0.05 to control case by Student's *t*-test.

To determine whether similar processes and ecology account for the chemical cycling observed in Guerrero Negro mats as in Elkhorn Slough mats, both GN-S and GN-I mats were subjected to microcosm-based studies. Experiments were conducted on cores of harvested mat cut down to the top 2 mm and placed in microcosm vials with site water and sealed. Reduced gases and fermentation products were collected at periodic intervals over the diel. Hydrogen gas and organic acids were produced only during the dark portion of the diel cycle with more of each produced in GN-I mats (Figure 2), with the relative abundance of organic acids consistently corresponding to: acetate > formate > propionate (Figure 2B). Additionally, two separate manipulation experiments were conducted to differentiate the influence of nitrogen fixation and oxygenic photosynthesis on nighttime fermentation. Ammonium chloride manipulations were intended to suppress the effect of nitrogenase and nitrogen fixation to examine the impact on fermentation rates. No significant difference was observed on net hydrogen and organic acid production between control and nitrogen replete treatments (Figures 2A,B), with the exception of a reduction in net formate production in GN-I mats. This is consistent with previous work conducted on Elkhorn Slough mats that showed that hydrogen production at night was largely uninfluenced by nitrogen fixation and constitutive fermentation was the main contribution to hydrogen production (Burow et al., 2012).

**Figure 3:**
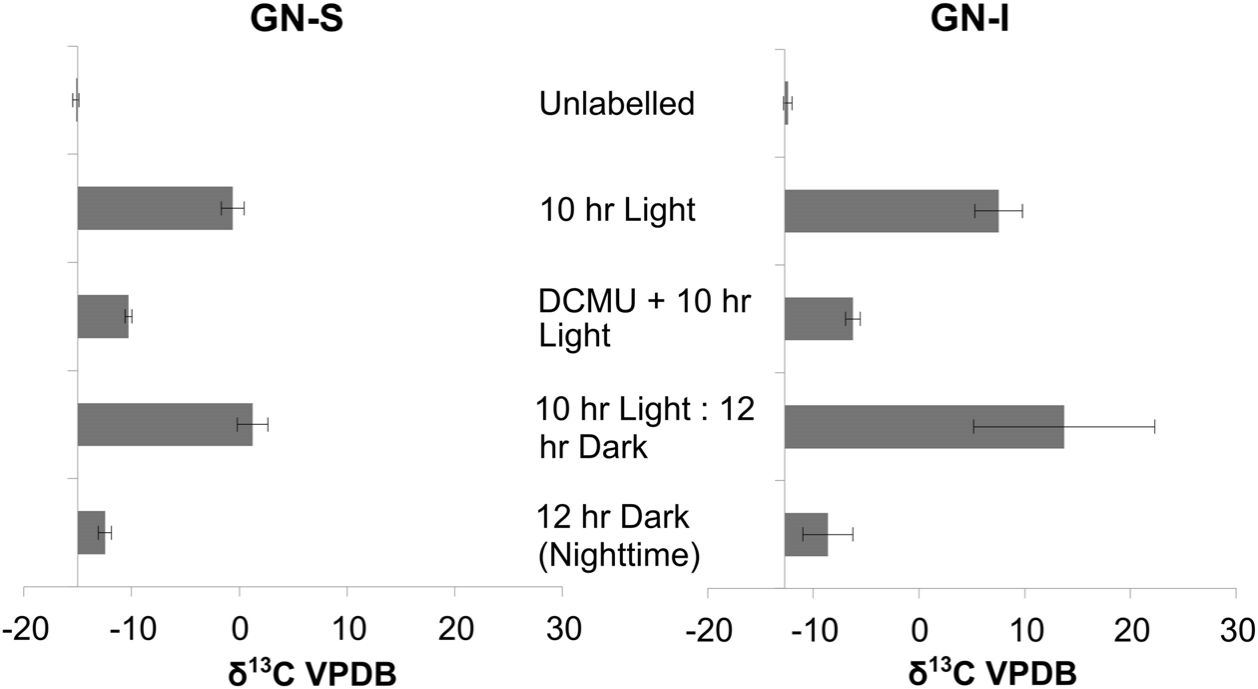
**Isotopic enrichment (measured by IRMS) of GN-S and GN-I mats following ^13^CHO_3_- labeling and DCMU inhibition of photosystem II and photoautotrophy**. Samples were collected after either 10 h of lighted label exposure, 10 h of light plus 12 h of darkness, or 12 h of darkness. Error bars are standard deviation of 4 replicate trials.

**Figure 4:**
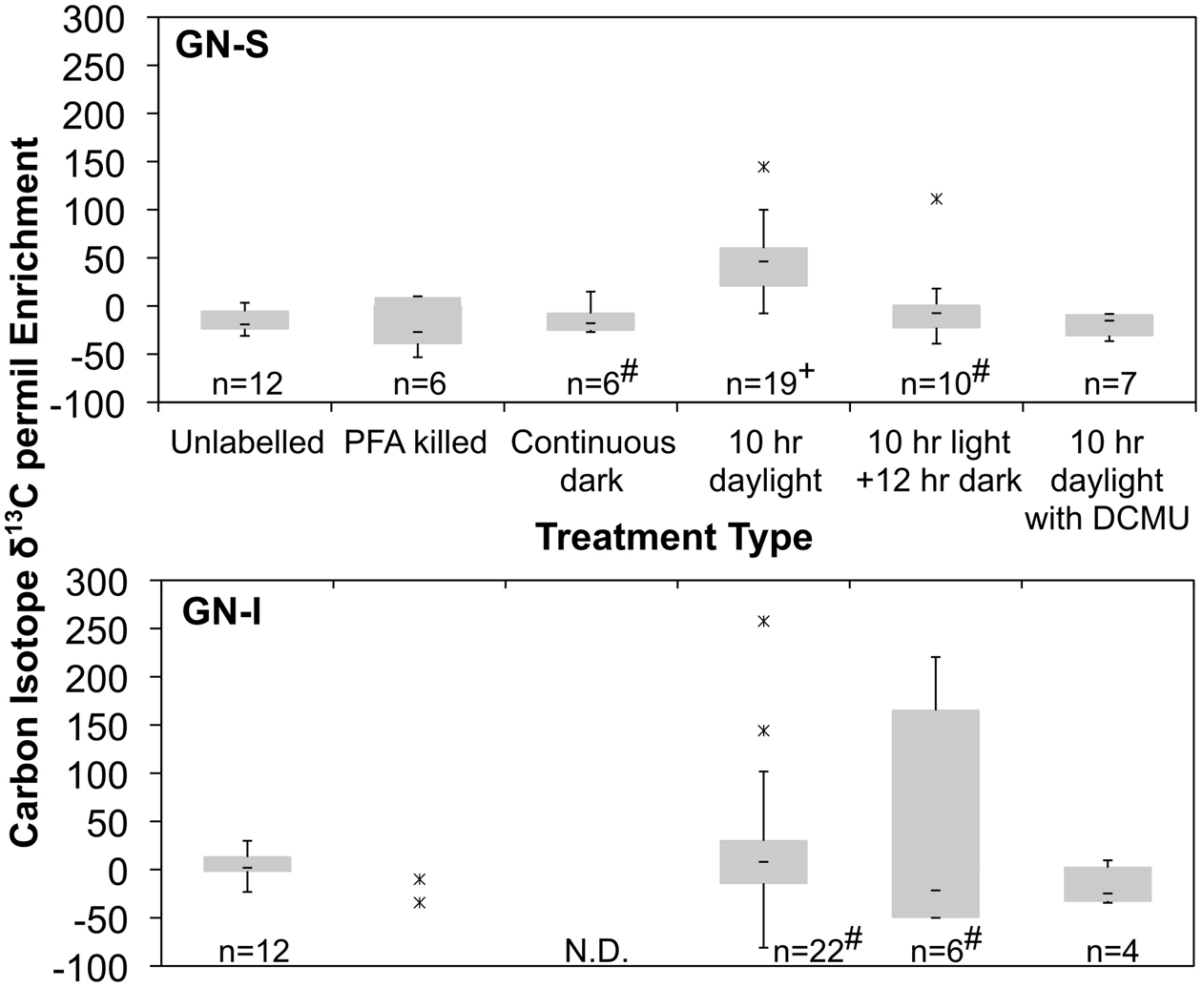
**Quartile box plots of NanoSIMS isotope ratio measurements of *Microcoleus* morphotype filaments labeled with ^13^C-bicarbonate for trials of unlabeled, PFA killed, continuous dark, 10 h daylight, 10 h daylight followed by 12 h night, and 10 h daylight with DCMU added**. Data was collected via multiple spot measurements of multiple filaments for each mat type (GN-S, at top; GN-I, at bottom). Asterisks are outlier datapoints identified as 1.5^*^IQR (Inter Quartile Range) outside quartiles. Outliers were retained in analysis. “#” denotes distributions for which the Shapiro–Wilk test *p* < 0.05. “+” denotes significantly different to all other GN-S trials (Wilcoxon test, *p* < 0.05). “N.D.” denotes no data taken.

In the second experiment, DCMU (which inhibits photosystem II found in *Cyanobacteria*) was added in an effort to help clarify the role of oxygenic photosynthesis on fermentation activity in these mats. Inhibition was verified by oxygen microelectrode profiles showing anoxic conditions within 200 μm of the mat water interface (data not shown). Net hydrogen production was significantly lower in DCMU treated mats (Figure 2C), while net acetic acid production was higher (Figure 2D). This was the only manipulation experiment in which hydrogen and net organic acid production did not change in concert in a microcosm. Figure 3 shows isotopic enrichment following ^13^C-bicarbonate labeling of GN mats exposed to DCMU and light. In both mat types, the ^13^C enrichment of DCMU-exposed mats resembled that of control mats incubated in the dark rather than light, suggesting that mats were not fixing carbon during the day. These observations are consistent with past studies that used DCMU to switch mats into an anoxic, nitrogen-fixing, sulfate-reducing mode during daylight (Bebout et al., 1987, 1993; Steppe and Paerl, 2002). We further verified our findings by measuring daytime ^13^C-bicarbonate uptake in individual *Microcoleus* morphotype filaments by NanoSIMS isotopic imaging (Figure 4). Control filaments incorporated significantly more labeled bicarbonate relative to DCMU-exposed filaments during daylight.

### Identifying major hydrogen producers and consumers in mats by hydrogenase (HOXH) phylogeny

**Figure 5:**
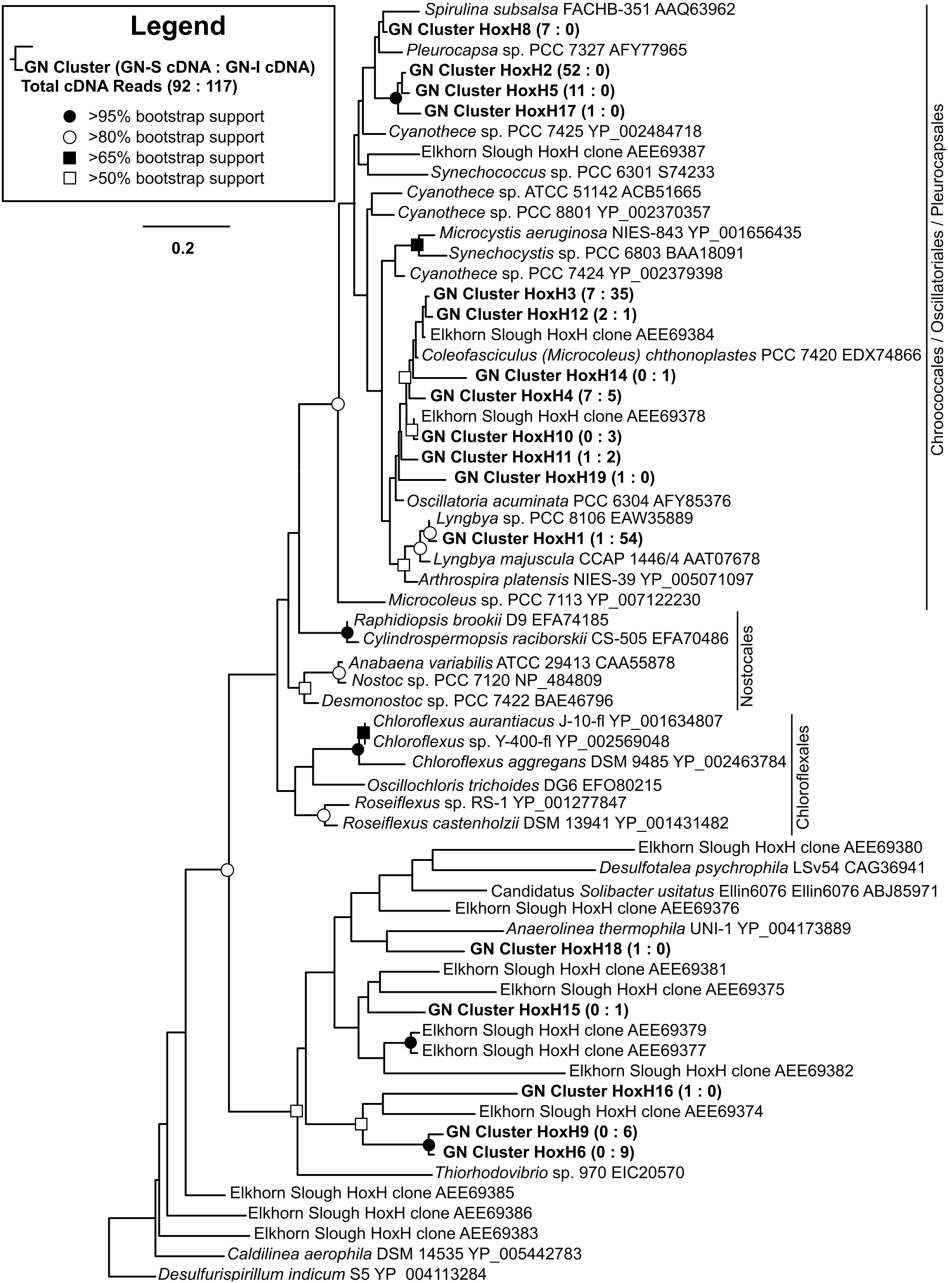
**Phylogenetic relationship of type 3b [NiFe]-hydrogenases detected from GN-I and GN-S mats**. Maximum likelihood (ML) phylogram inferred from partial *hoxH* transcript sequence data for selected taxa, with an emphasis on the *Cyanobacteria* and *Chloroflexi*. Symbols denoting bootstrap support values are for both ML and neighbor joining analyses. Representative sequences for OTUs identified from this study are in bold. Numbers in parentheses after study sequences denote number of sequences for each OTU from GN-S mats (left), and GN-I mats (right).

To identify the organisms responsible for the production and consumption of hydrogen in Guerrero Negro mats, 117 transcripts (in 17 OTUs) of [NiFe]-hydrogenases of type 3b were sequenced and passed quality control. Figure 5 shows the phylogenetic relationship of OTUs detected from GN-I and GN-S mats. In both mat types, the majority of the observed hydrogenases were from *Cyanobacteria* of both filamentous and unicellular types. In GN-I mats, transcript OTUs related to *Lyngbya* sp. PCC 8106 were seen to be most abundant, and additional OTUs of mat-associated filamentous *Cyanobacteria* such as *Microcoleus chthonoplastes* PCC 7420 were also seen. In GN-S mats, the most abundant OTUs came from an unknown cyanobacterium in *Oscillatoriales* or *Pleurocapsales* whose closest BLAST match (~94% ID by BLASTp) was *Pleurocapsa* sp. PCC 7319. Transcripts related to *Microcoleus chthonoplastes* PCC 7420 were detected as well. The remaining hydrogenases observed in these mats were most related to clusters of hydrogenases previously reported in Elkhorn Slough mats (Burow et al., 2012). Therefore, the distribution of abundant hydrogenases in hypersaline microbial mats appears to fit a pattern of cyanobacterial clusters, with additional diverse unidentified groupings of lower abundances. Notably, no sequences for hydrogenases from the anoxygenic phototrophs of the *Chloroflexi* were detected.

### Accumulation of fixed carbon and release of fermentation byproducts in microcosm studies

**Figure 6:**
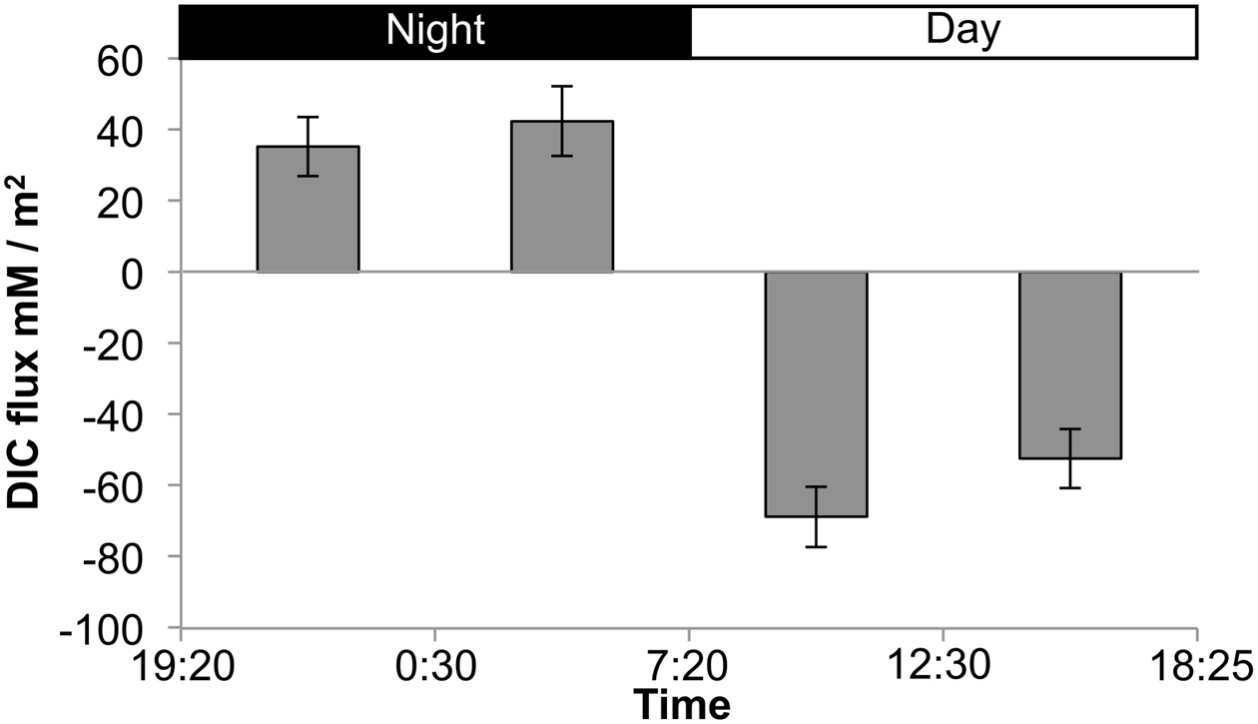
**Flux chamber measurements of dissolved inorganic carbon (DIC) flux from GN-I mats over a diel period**. Values reflect net flux of DIC between the indicated times. Error bars are standard deviations of 4 replicate trials.

**Figure 7:**
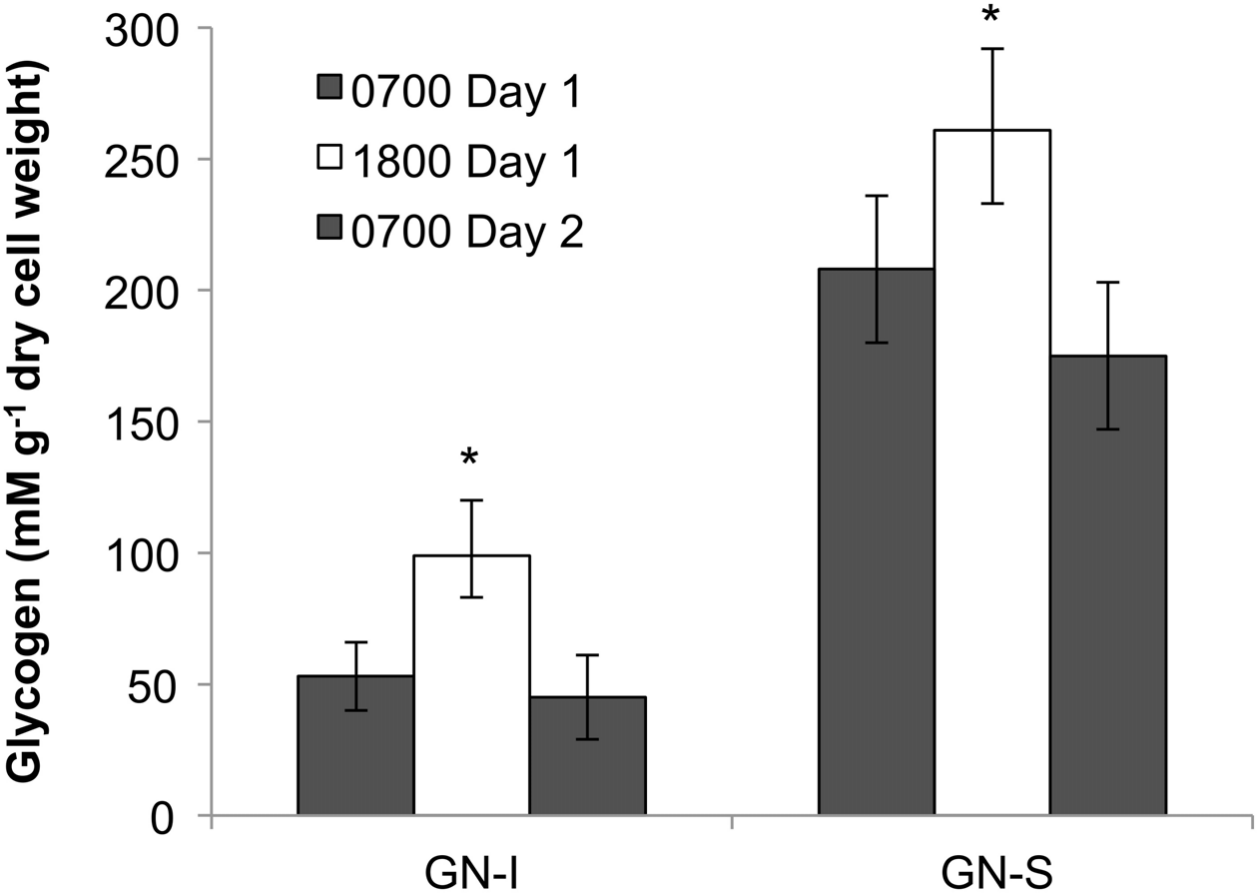
**Glycogen accumulation over the diel cycle in GN-I and GN-S mats over the diel**. Error bars represent standard deviation of 9 replicate cores per time point. Asterisk indicates *p* < 0.05 by unpaired 2-tailed Student's *t*-test to both 0700 sample sets.

The presence of fermentation products in Guerrero Negro mat types, though detectable, may not be in meaningful quantity to influence the overall cycling of carbon in the ecosystem. Therefore, several experiments were completed to measure the relative magnitude of fixed carbon in relation to fermentation products released in GN-I mats. Changes in concentration of DIC in the water overlying incubated mats were taken to reflect total flux of carbon due to fixation or respiration (Figure 6). The observed flux was into the mats (negative) primarily during the daylight period and into the water column (positive) primarily during the nighttime period, consistent with previous work (Des Marais, 1995). Glycogen measurements from mat cores at different times were used to estimate the amount of stored fixed carbon in mats across the diel. Both mat types did exhibit a daytime accumulation (net gain) and nighttime loss (net loss) of glycogen, though the absolute magnitude of glycogen was 2.5-fold higher in GN-S mats than in GN-I mats while the net diel change differed by only 25% (higher in the GN-S mat) (Figure 7).

To relate these different fluxes and pools to each other, and to the fermentation results from the microcosm studies, an electron mass balance was constructed (Table 2) (See Section Photosynthesis/Fermentation Mass and Electron Balance for methods and conditions used). DIC and glycogen fluxes over the day and night period were averaged to determine a single flux over the diel. For organic acids and hydrogen, both “net” and “total” values are given. “Net” values were derived from fluxes observed under control conditions, and were assumed to incorporate the effects of H_2_ consumption by spatially-associated accessory populations as well as H_2_ production in whole intact mats. “Total” values were derived from fluxes observed when mats were homogenized to disrupt physical associations and thereby decrease or eliminate consumption by accessory organisms. Under these conditions, the flux of hydrogen, acetate, and propionate roughly doubled over controls. Because it is not certain that disruption completely eliminates consumption of fermentation products, this “total” value should be taken as a lower bound on the maximum possible production of fermentation products. Approximately 81% of the photosynthetically fixed electrons are released as total fermentation products during the night. Based on the differing “net” and “total” fluxes of fermentation products, it appears that about 41% of net photosynthetic carbon fixation was ultimately consumed by accessory populations within the mat. Quantitatively, hydrogen was a minor contributor to the overall cycling of photosynthetically-fixed electrons in these mats. The majority was accounted for as organic acids, specifically as acetate.

**Table 2:** **Fermentation product concentration and equivalent electron balance in GN-I mat microcosms showing the amount of light captured electrons in fixed carbon analytes (during the day) and in fermentation by-products (produced at night)**.

	**Daytime flux/storage**	**Nighttime flux/storage**	**Avg. diel flux/storage**	**e^−^ equivalents**	**e^−^ flux/storage**
	**mmol/m^2^ Avg. (*SD*)**	**mmol/m^2^ Avg. (*SD*)**	**mmol/m^2^ Avg. (*SD*)**		**mmol e^−^/m^2^**
Fixed inorganic-C (CH_2_O)	−121.4 (10.3)	77.5 (6.9)	99.5 (8.6)	4	397.9 (34.4)
Glycogen	21.2 (25.2)	−31.6 (30.4)	26.4 (27.8)	24	633.8 (667.2)
**NET FERMENTATION**					
Acetate	–	14.9 (0.9)	14.9 (0.9)	8	119.2 (7.5)
Formate	–	8.3 (1.2)	8.3 (1.2)	1	8.3 (1.2)
Propionate	–	2.4 (0.5)	2.4 (0.5)	13	31.7 (6.1)
H_2_	–	0.83 (0.05)	0.83 (0.05)	2	1.66 (0.09)
**TOTAL FERMENTATION**					
Acetate	–	28.0 (1.3)	28.0 (1.3)	8	224.2 (10.1)
Formate	–	10.0 (1.0)	10.0 (1.0)	1	10.0 (1.0)
Propionate	–	6.6 (1.2)	6.6 (1.2)	13	85.3 (15.1)
H_2_	–	1.77 (0.18)	1.77 (0.18)	2	3.55 (0.36)
% Fixed e^−^ fermented					81.2 (14.6)
% Fixed e^−^ taken up by accessory microbes					40.8 (14.0)

*Values were normalized to microcosm mat diel flux or storage rate and core cross-sectional area, mmol/m^2^. Net fermentation by-product measurements were from control microcosms, and total fermentation byproducts were from homogenized microcosms*.

### Microcosm studies of hydrogen consumption in microbial mats

**Figure 8:**
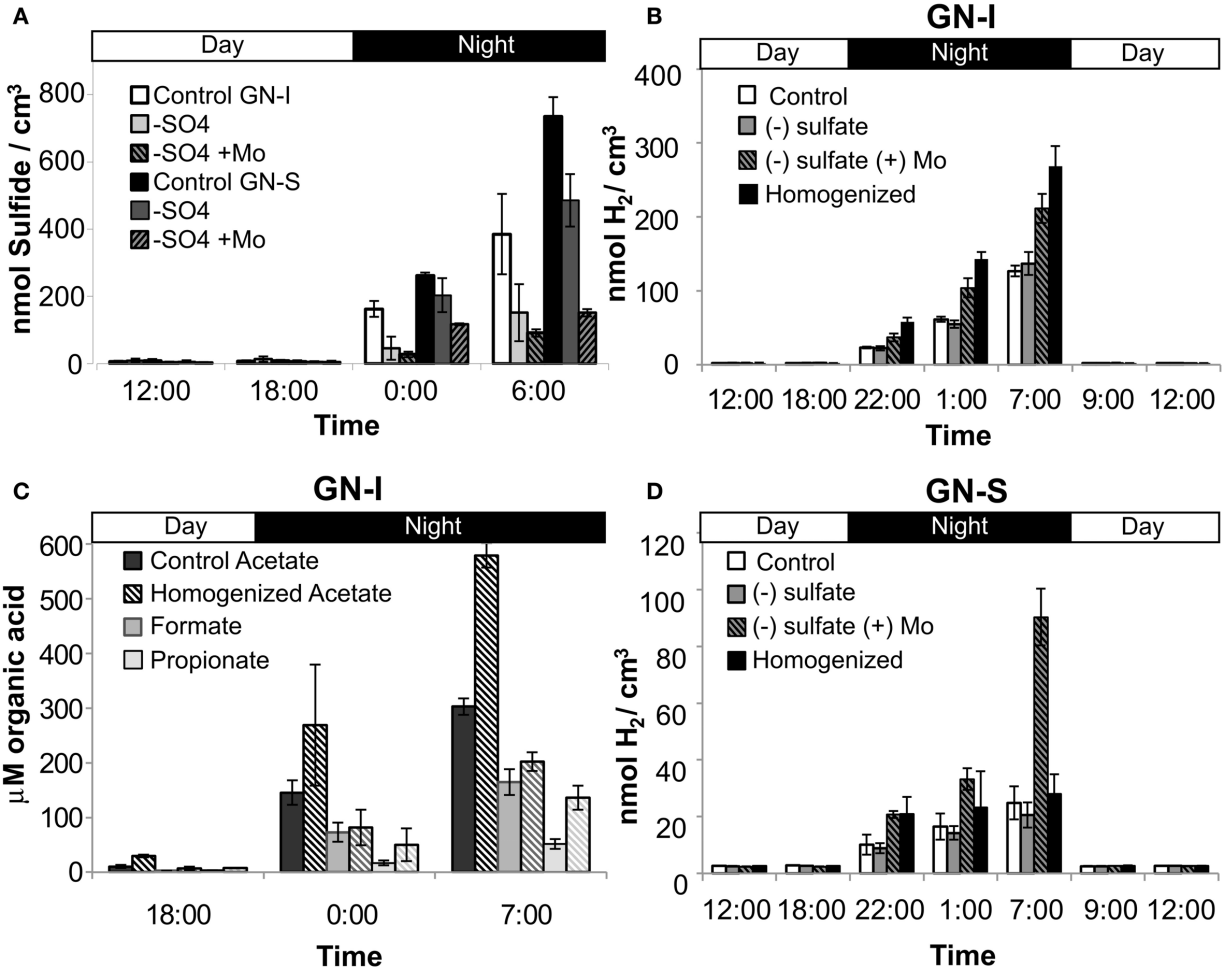
**Hydrogen consumption and SRB activity in two microbial mat types from Guerrero Negro, B.C.S., Mexico**. Net hydrogen sulfide production (both mats) **(A)**, net hydrogen production in GN-I mats **(B)**, net organic acid production in GN-I mats **(C)**, and net hydrogen production in GN-S mats **(D)**, showing the effect of physical disruption, sulfate deprivation, and molybdate addition on microcosms. Error bars indicate standard deviation of 6 replicate trials.

Similar to the microcosms used to test nitrogen fixation and oxygenic photosynthesis in hydrogen production, a combination of experimental microcosms were used to characterize and quantify microbial hydrogen consumption. Sulfate deprivation and molybdate addition were intended to reduce the activity of sulfate reduction, and were conducted both individually and together in microcosms. Disruption was intended to physically separate mat community members and was conducted in a separate microcosm experiment. Both sulfate deprivation alone, and sulfate deprivation with molybdate addition resulted in significantly diminished accumulation of hydrogen sulfide (the end product of sulfate reduction) within microcosm vials, but with the second case having a much larger effect (Figure 8A). In GN-I mats, net hydrogen production was enhanced by both disruption and molybdate addition with sulfate deprivation (Figure 8B), while hydrogen production in GN-S mats was enhanced only by sulfate deprivation with molybdate inhibition (Figure 8D). Because our method for quantifying organic acids is not compatible with molybdate, we could only examine the effect of physical disruption on organic acid production, which showed that production of acetate and propionate roughly double in GN-I mats due to physical disruption (Figure 8C).

### Organic acid uptake in microbial mats

**Figure 9:**
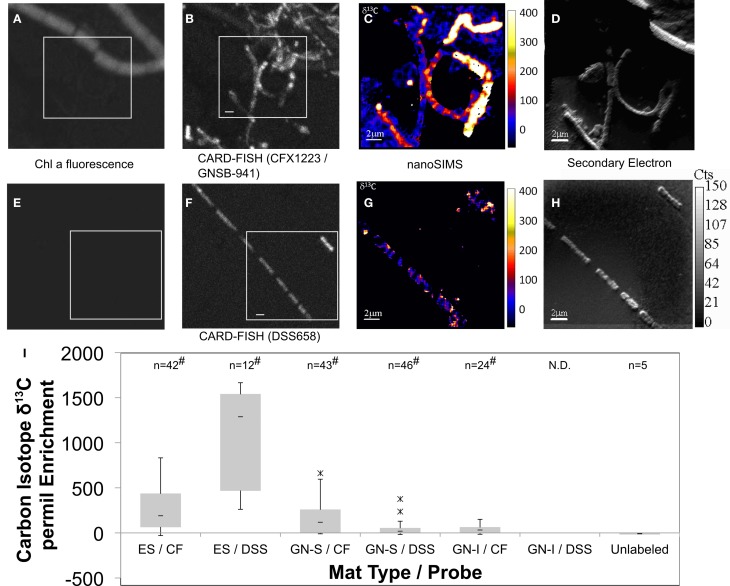
**Paired Chlorophyll *a* natural fluorescence, confocal, CARD-FISH, and ^13^C-acetate NanoSIMS (permil) micrographs of GN-S mat (A–H)**. *Chloroflexi* (CFX1223 and GNSB-941) **(A–D)** and *Desulfosarcina*/*Desulfobacteraceae* (DSS658) hybridized filaments **(E–H)** shown in conjunction with Chlorophyll *a* natural fluorescence, CARD-FISH, ^13^C enrichment, and secondary electron images, respectively. Bar represents 2 μm. Quartile box plots of ^13^C-acetate NanoSIMS spot measurements from Elkhorn Slough (ES), GN-S, and GN-I mats shown in **(I)** with significant difference test comparison to isotopic spot measurements on microbial filaments showing no hybridization to either probe. Asterisk denotes outliers 1.5^*^IQR outside quartiles (though still kept for analysis). “#” denotes both Shapiro–Wilk test *p* < 0.05 and Wilcoxon difference test *p* < 0.05 (compared to unhybridized filaments). “N.D.” indicates no hybridized filaments were detected. ES denotes Elkhorn Slough mat samples, CF denotes CFX1223 and GNSB-941 probes, and DSS denotes DSS658 probes.

Organic acids produced by nighttime fermentation have the potential to serve as substrates for both sulfate reduction and photoheterotrophy. The former was suggested by the significant enhancement of nighttime organic acid production upon physical disruption (Figure 8C). Photoheterotrophy was suggested in Elkhorn Slough mats by Burow et al. (2013) to be related to the activity of *Chloroflexi*. To further characterize and quantify organic acid uptake, mats from both GN and Elkhorn Slough were incubated overnight with ^13^C-labeled acetate, and analyzed for isotopic enrichment by NanoSIMS. The final amended ^13^C-acetate concentration (0.2 mM) was not greater than maximum values of accumulation in mats and represented similar concentrations in natural unamended mats. Probes specifically for phylum *Chloroflexi* (CFX1223 and GNSB-941) and for *Desulfobacteraceae* (DSS658) were used to determine if hybridized cells took up acetate. Figure 9 shows NanoSIMS measurements of hybridized filaments for three mat types compared to microbial filaments with no hybridization. Figures 9A–D show paired Chlorophyll *a* autofluorescence (A), *Chloroflexi* probe CARD-FISH (B), and NanoSIMS ^13^C enrichment (C), and NanoSIMS secondary electron images (D). Figures 9E–H shows a similar set of paired images for the *Desulfobacteraceae* probe. Figure 9I shows a quartile box plot summarizing NanoSIMS isotopic spot measurements over a number of different mat types and across replicate samples and the significance of the distribution when compared to filaments that did not hybridize with probes. All three mat types showed enrichment in CFX1223/GNSB-941 hybridized cells by NanoSIMS imagery and spot measurements, but varying patterns of enrichment between filaments within samples was observed. DSS658 hybridized filaments also showed enrichment, but generally much less than for CFX1223/GNSB-941 filaments. In GN-I mats, no DSS658 hybridization was visible in microscopy. In all cases, significant enrichment was observed over unhybridized filaments, but enrichment in *Chloroflexi* and *Desulfobacteraceae* was notably higher in Elkhorn Slough samples.

### Surveying the diversity of sulfate reducing bacteria through DsrA phylogeny in guerrero negro mats

**Figure 10:**
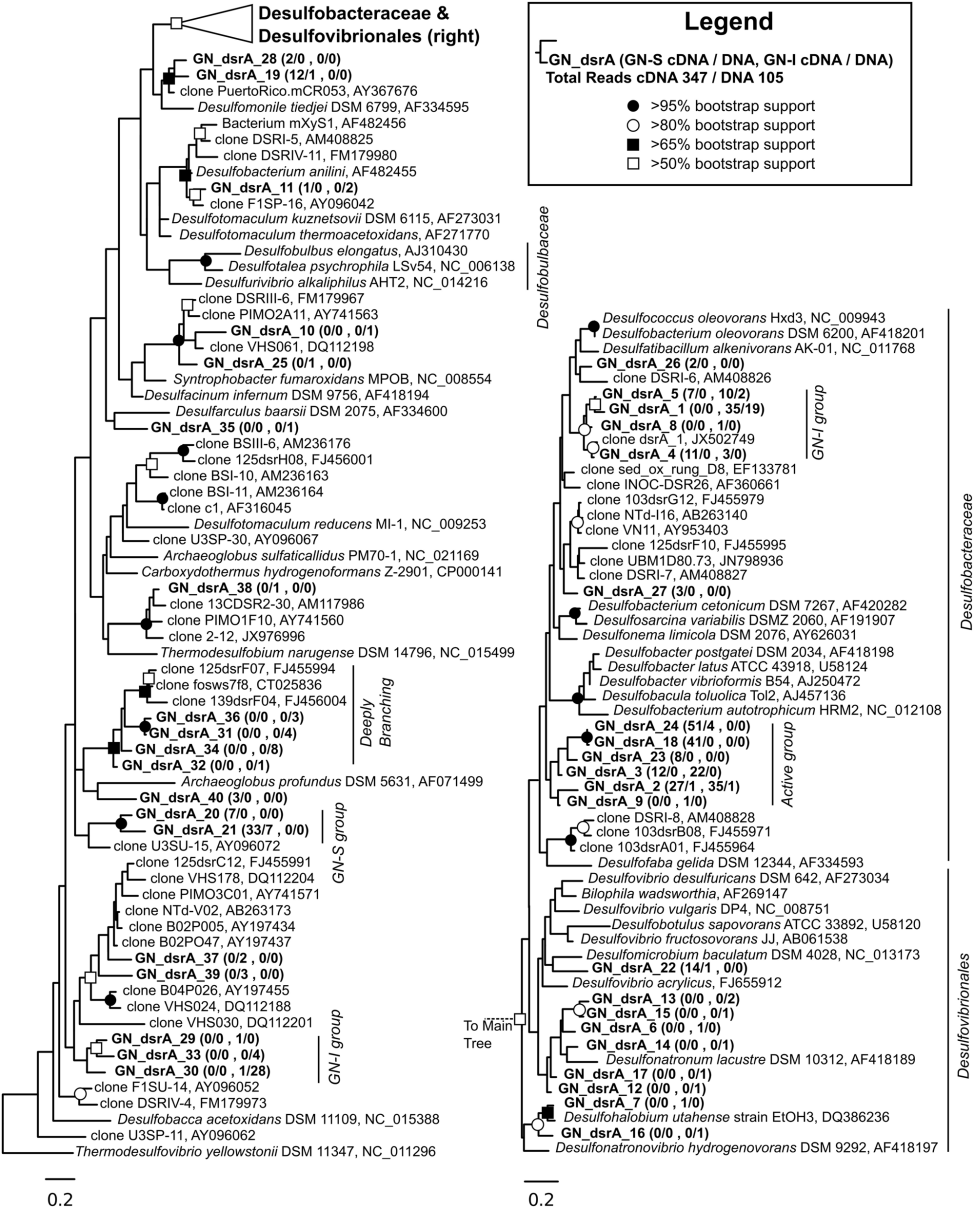
**Phylogenetic relationship of translated *dsrA* genes and transcripts detected from GN-I and GN-S mats**. Maximum likelihood phylogram inferred from partial DsrA sequence data for selected taxa. Symbols denoting bootstrap support values are for both ML and neighbor joining analyses. Representative sequences for OTUs identified from this study are in bold. Numbers in parentheses after study sequences denote number of sequences for each OTU from GN-S mats (first), and GN-I mats (last), with ratios for cDNA/DNA reads shown, respectively.

DsrA phylogeny of sequences derived from both mat types are shown in Figure 10. Nodes of the family *Desulfobacteraceae* did not generally bootstrap well, but genera of the family *Desulfobacteraceae* formed a monophyletic clade, as did genera within the family *Desulfobulbaceae*. The constructed phylogeny was consistent with the work of Leloup et al. (2009) which showed *Desulfovibrionales* affiliating with *Desulfobulbaceae* within *Desulfobacterales* and also identified both the orthologous and xenologous *Desulfotomaculum*. In total, 347 cDNA and 105 DNA reads were obtained and binned into 40 OTUs for *dsrA* genes from the GN-I and GN-S mats. The maximum likelihood analysis for the translated *dsrA* data in Figure 10 revealed that OTUs were distributed throughout the phylogeny. However, the majority of sequences were clustered into five main groups: two within the *Desulfobacteraceae* and three within the deeply branching regions of the tree. Though DSS658 labeled cells could not be found in GN-I mats by CARD-FISH, *dsrA* genes and transcripts belonging to *Desulfobacteraceae* were detected in both mat types. One main cluster, primarily from the GN-I mats, formed a well-supported clade with a DsrA sequence identified from the intertidal mats found at Elkhorn Slough, CA, USA (accession JX502749, Burow et al., in press), this may represent a novel “intertidal” lineage of *Desulfobacteraceae*. A second cluster did not affiliate closely with any described SRB taxa, and was not supported by bootstrap analysis, but was placed within the *Desulfobacteraceae*. This grouping was particularly high in transcript content (197 cDNA reads vs. only 6 DNA reads) and distributed in both GN-I and GN-S environments. Finally, several OTUs grouped deep in the tree together in what Harrison et al. (2009) refers to as the “deep-branching *dsrA*.” These OTUs tended to cluster with other cloned sequences from studies of sediments and marshes (Castro et al., 2002; Bahr et al., 2005) and have an as yet unknown role in these mats. No naming convention for clades was apparent in the numerous published DsrA phylogenies, but these deep OTUs affiliated with sequences belonging to what have been previously termed clade IV (Dhillon et al., 2003; Bahr et al., 2005; Zhang et al., 2008), clade V (Kaneko et al., 2007; Zhang et al., 2008; Harrison et al., 2009), and/or clade DSR-2 (Castro et al., 2002) and at least one clade may contain members derived from horizontal gene transfer (Mussmann et al., 2005). Of the deeply-branching clades, one deeply branching group contained an abundant OTU from GN-S, one contained an abundant OTU from GN-I, and the last contained rare OTUs from GN-I mats.

## Discussion

Previous work (Skyring et al., 1989; Burow et al., 2012) suggested that nighttime production of reduced gases results from photoautotrophy and storage of reduced carbon by *Cyanobacteria* with subsequent fermentation of stored photosynthate following the onset of dark, anoxic conditions (Hoehler et al., 2001; Des Marais, 2003). In Elkhorn Slough mats, *Cyanobacteria* were indicated as the dominant fermenter (Burow et al., 2012) and sulfate reducers as a key consumer of H_2_ (Burow et al., in press). This study builds on previous reports of H_2_ cycling in microbial mats in three important regards. First, bulk chemical cycling and the underlying ecology are shown to be common features of geographically diverse mats, as well as mats constructed by distinctly different *Cyanobacteria*. Second, organic acid cycling is characterized and quantified, and shown to represent a significant component of overall carbon and electron flow in the studied mats. Last, multiple methods are utilized to demonstrate that exchange of fermentation products serves to directly link *Cyanobacteria* with sulfate reducers and anoxygenic phototrophs.

### Fermentation is quantitatively important in mats

If daytime oxygenic photosynthetic fixation of carbon drives the subsequent nighttime fermentation to hydrogen and organic acids, a series of changes in metabolites should be observable as: (1) accumulation during the day and release at night of inorganic carbon based on measurements of the flux of dissolved inorganic carbon (DIC) across the mat-water interface, (2) accumulation during the day and depletion at night of small stored carbon polymers within the mat (e.g., glycogen), and (3) a rise in organic acid flux within the mat at night. These were all observed in the studies presented here. As shown in Table 2 and Figure 6, inorganic carbon was incorporated into mats during daytime. Figure 7 indicated glycogen as the primary fixed carbon storage molecule in hypersaline microbial mats, with GN-S mats accumulating much more glycogen. Approximately ~81% of all carbon fixed during the day in GN-I mats was subsequently fermented at night, with most of the net accumulation of fermentation products occurring as organic acids rather than as hydrogen. The small amount of hydrogen released by fermentation activity relative to organic acids was unsuspected given that the hydrogen concentrations in the mat go through a four order of magnitude change in concentration throughout a diel cycle and particularly given that net hydrogen fluxes in GN-I mat are 10 times greater than in the GN-S mat. A stoichiometric fermentation of glucose to acetic acid, carbon dioxide, and hydrogen would produce nearly 2:1 electron ratios of acetate and hydrogen, but the ratios measured in this study were typically closer to 100:1 (Table 2) and suggests that organic acids provide the most quantitatively observable flux of reductant and energy available to the broader community of microbes within the mat under dark/anoxic conditions.

### Constitutive fermentation by *Cyanobacteria* was responsible for a majority of hydrogen and organic acid production

The results of this study indicated similar diel patterns in organic acid and hydrogen production in hypersaline microbial mats from different locations and of different types. Because fermentation of photosynthate represents a loss of reducing power and delivers the lowest energy yield among potential catabolic processes, it could be viewed as a process to be minimized (that is, to be employed only if required by the demands of nighttime metabolism). One potentially large demand for such fermentation would be the energy required to fuel dinitrogen fixation and, for this reason, we examined whether inhibition of dinitrogen fixation (by addition of ammonium as a source of combined nitrogen) diminished the yield of fermentation products. As shown in Figure 2A, and consistent with previous observations in Elkhorn Slough, ammonium addition does not appear to affect fermentation. This suggests that fermentation may be a constitutive aspect of metabolism in these mats, rather than being regulated in response to energetic or other demands of metabolism. The glycogen data and relative ratios of organic acid flux indicate that fermentation of glycogen to acetate was the dominant nighttime pathway, with a smaller component of mixed acid fermentation.

Photosynthetically-fixed carbon was indicated to be the feedstock for fermentation. In GN-I mats, enhanced levels of bicarbonate incorporation (Figure 3) were reflected in significantly higher net efflux of fermentation products than in GN-S mats (Figure 2). Indeed, when DCMU was used to limit oxygenic photosynthesis, net fermentation dropped more than in GN-S mats, indicating oxygenic photosynthesis in *Cyanobacteria* was the most likely cause behind net fermentation productivity of these mats. At the same time, NanoSIMS measurements of bicarbonate incorporation (Figure 4) show increased accumulation of label in GN-I *Microcoleus* filaments over GN-S filaments. These findings point to the accumulation of glycogen being more common in GN-S mats, but increased fixation and increased catabolic metabolism being more common in GN-I mats and fits the observation that GN-I mats are adapted to exist in a dynamic turbulent intertidal zone and GN-S mats are adapted to quiescence (Bebout et al., 1994).

Pyrotag assays (Figure 1) corroborated previous reports of the abundance (approximately one fourth of DNA sequences from pyrotag libraries) of *Chloroflexi* in GN-S mats (Ley et al., 2006) as well as a similar level of *Chloroflexi* pyrotags seen in GN-I mats. Moreover, previous studies have shown that anoxygenic phototrophy was found to account for 10–40% of carbon fixation in GN-S mats (Finke et al., 2013), assumed to be by phototrophic sulfide oxidation. It has also been demonstrated, in the case of hot springs microbial mats that filamentous photoautotrophic *Chloroflexi* can have a role in fermentation, including hydrogenase transcript expression at night (Klatt et al., 2013), or a role indirectly driving fermentation (Otaki et al., 2012). Yet, in this study anoxygenic phototrophy appears to play only a minor role in nighttime fermentation in Guerrero Negro mats. Expression ratio (cDNA:DNA pyrotags) data demonstrated that *Cyanobacteria* (specifically genus *Microcoleus* in GN-S mat and both *Microcoleus* and genus *Lyngbya* in GN-I mat) maintain a consistent level of ribosomal expression between day and night and at a level much higher than any other phylogenetic group detected. The only hydrogenases attributable to phototrophs that were expressed at night (Figure 5) were associated with *Cyanobacteria*; no type 3b [NiFe]-hydrogenases from any anoxygenic phototrophic *Chloroflexi* group were recovered. However, given that marine filamentous anoxygenic phototrophs are diverse and mostly uncharacterized in Guerrero Negro mats (Nübel et al., 2001; Ley et al., 2006) the phylogeny of novel *Chloroflexi* hydrogenases present in these systems is an avenue of future study. But overall, given the dominance of several types of *Cyanobacteria* in pyrotags and hydrogenase transcripts, *Cyanobacteria* were likely the metabolically dominant phototrophic fermenters in mats. Interestingly, the HoxH tree (Figure 5) does suggest that a *Cyanobacteria* other than *Microcoleus chthonoplastes* PCC 7420 was the main hydrogen producer in GN-S mats and that different species of *Cyanobacteria* may differ in their capacity for hydrogen production.

Under natural conditions, both the GN mats and Elkhorn Slough mats (Burow et al., 2012) were characterized by net fluxes of hydrogen and organic acids out of the mats at night due to fermentation activity. However, in mats incubated with DCMU for the previous photoperiod, net hydrogen fluxes were reduced relative to the unamended treatments, whereas the flux of organic acids out of the mats was not significantly different (Figures 2C,D), This differential effect of DCMU on hydrogen vs. organic acid flux was not previously observed in Elkhorn Slough samples (Burow et al., 2012). Overall, though the DCMU in mats has been shown to inhibit photosystem II in *Cyanobacteria* and the establishment of anoxic conditions, the response of whole mat communities to this photosystem shutdown in daylight was variable across different mat types and is still poorly understood, especially with respect to the daytime sulfide cycling. Mechanisms in both *Cyanobacteria* and in *Cyanobacteria*-associated members, such as phototrophic sulfide oxidation, may be acting in concert to alter both the cycling of hydrogen as well as the original production of hydrogen.

### Hydrogen and organic acid availability leads to uptake by *Cyanobacteria*-associated microbes in hypersaline microbial mats

Release of cyanobacterial fermentation products within the closely packed matrix of the mat offers a flux of potential substrate to a range of terminal metabolizers. We hypothesized that SRB were the primary consumers of hydrogen and organic acids under dark, anoxic conditions, due to the abundance of sulfate. Like in hot spring microbial mats (Otaki et al., 2012) they were suspected to require close physical proximity for hydrogen uptake. In GN-I mats, inhibition with molybdate significantly increased accumulation of hydrogen at quantitatively similar levels to physical disruption (Figure 8B). This was consistent with previous findings in the hypersaline microbial mats of Elkhorn Slough (Burow et al., 2012), and suggests that a physical association between *Cyanobacteria* and SRB underlies most of the observable consumption of fermentation products within these mats (Burow et al., in press). Molybdate also enhanced accumulation of hydrogen in the GN-S mats, but physical disruption in those mats did not result in significantly greater net hydrogen flux relative to controls (Figure 8D). Thus, while SRB appear to be the dominant sink for fermentation products under dark conditions in GN-S mats, physical associations appear to be less important than in the GN-I and Elkhorn Slough mats, though there is evidence that physical proximity could still be necessary (Fike et al., 2008). However, the failure of physical disruption techniques to separate mat members apart in GN-S mats cannot be discounted, nor can the possibility of unique motile SRBs in GN-S mats be discounted.

We show here that disruption of the GN-I microbial mat, with a presumed separation of diverse members of the mat community from *Cyanobacteria*, led to a proportionally greater increase in the flux of organic acids, relative to hydrogen. This was consistent with the idea that organic acid consumption was also dependent (and possibly even more dependent than hydrogen) on tight physical association between producing and consuming organisms. Preliminary efforts were made to identify organisms that may be consuming acetate, via NanoSIMS analysis of samples incubated with ^13^C-labeled acetate under dark/anoxic conditions. NanoSIMS analysis (Figure 9) confirmed that filamentous members of *Chloroflexi* and *Desulfobacteraceae* were significant consumers of acetate at night and may be important members of these close spatial associations, though no known filamentous SRB could be identified in DsrA phylogenetic analysis. Pyrotags of *Proteobacteria* show that purple non-sulfur bacteria were also quantitatively important, particularly in the GN-I mats (Figure 1B). The present study did not specifically investigate acetate uptake by these organisms. However, the diverse and robust nature of the metabolism within this group (with various members being able to conduct autotrophic, heterotrophic, photosynthetic, chemotrophic, aerobic, and anaerobic metabolisms) suggests that they should also be examined for significant nighttime consumption of fermentation products in GN-I mats.

## Conclusion

In this study, a suite of methods identified that a variety of *Cyanobacteria* were the dominant fermentive organism responsible for hydrogen production during nighttime constitutive metabolism. Furthermore, hydrogen production was driven by daytime carbon storage, and total hydrogen produced was a fraction of the total fermentation potential, with the majority of fermentation products being organic acids (especially acetate). This work also identified uptake of acetate during nighttime by both sulfate reducing bacteria and filamentous *Chloroflexi* provided an important linkage to *Cyanobacteria*. Taken together, these results indicate the nighttime fermentation of stored light energy can explain the close association of the filamentous *Chloroflexi* and of the SRB with cyanobacterial filaments.

### Conflict of interest statement

The authors declare that the research was conducted in the absence of any commercial or financial relationships that could be construed as a potential conflict of interest.
