# Supplementary material for: Fermentation couples Chloroflexi and sulfate-reducing bacteria to Cyanobacteria in hypersaline microbial mats
Source: Front Microbiol. 2014 Feb 26;5:61. doi: 10.3389/fmicb.2014.00061 (PMC3935151; doi:10.3389/fmicb.2014.00061)
Supplement: Supplementary file 1 [file Krona_charts_supplemental.zip › OTU table krona/GNI_LY_1200_cDNA_otutable.html]

Javascript must be enabled to view this page.

magnitude
 1.00000000000002
 .973780911709231
 .000148972092561327
 9.93147283742179e-05
 9.93147283742179e-05
 0
 0
 0
 4.96573641871089e-05
 4.96573641871089e-05
 0
 .00288012712285232
 .00288012712285232
 0
 0
 0
 0
 .00273115503029099
 .00248286820935545
 .00208560929585858
 0
 .000148972092561327
 0
 0
 0
 0
 0
 0
 0
 0
 .000148972092561327
 4.96573641871089e-05
 0
 0
 0
 0
 0
 .0269639487536002
 .000148972092561327
 .000148972092561327
 0
 .000297944185122654
 .000297944185122654
 .000248286820935545
 0
 0
 .0182739100208561
 .018224252656669
 .00839209454762142
 .000546231006058198
 .000347601549309763
 0
 0
 0
 0
 0
 .00342635812891052
 0
 0
 0
 0
 .00377395967822028
 .00377395967822028
 .00203595193167147
 9.93147283742179e-05
 4.96573641871089e-05
 0
 0
 0
 .000148972092561327
 4.96573641871089e-05
 4.96573641871089e-05
 4.96573641871089e-05
 0
 0
 .000148972092561327
 0
 0
 .00446916277683981
 .00446916277683981
 .00322772867216208
 .000198629456748436
 4.96573641871089e-05
 0
 4.96573641871089e-05
 0
 0
 0
 0
 .000198629456748436
 .000198629456748436
 .000198629456748436
 .000198629456748436
 4.96573641871089e-05
 0
 0
 0
 0
 0
 9.93147283742179e-05
 0
 0
 9.93147283742179e-05
 9.93147283742179e-05
 4.96573641871089e-05
 .0436488231204687
 .00729963253550502
 .00729963253550502
 .00486642169033668
 .00124143410467772
 .000446916277683981
 0
 .000794517826993743
 .000794517826993743
 .000347601549309763
 0
 .03555467275797
 .0340649518323567
 .00297944185122654
 9.93147283742179e-05
 .0241831363591221
 .00918661237461516
 .00695203098619526
 .00144006356142616
 .00104280464792929
 .000893832555367961
 .000844175191180852
 0
 0
 0
 .819743768000802
 .819743768000802
 .00864038136855696
 .00804449299831165
 .000397258913496872
 0
 0
 0
 .00456847750521402
 4.96573641871089e-05
 0
 .000446916277683981
 0
 0
 0
 .000347601549309763
 .000297944185122654
 9.93147283742179e-05
 0
 4.96573641871089e-05
 0
 0
 0
 .000446916277683981
 .000297944185122654
 .000198629456748436
 9.93147283742179e-05
 4.96573641871089e-05
 4.96573641871089e-05
 4.96573641871089e-05
 0
 .809514350978257
 .807478399046586
 .00188697983911014
 0
 0
 .646141622802665
 .512066739497468
 .0094348991955507
 4.96573641871089e-05
 .0468268944284437
 .0239845069023736
 .00139040619723905
 0
 .000148972092561327
 0
 4.96573641871089e-05
 4.96573641871089e-05
 0
 .063362796702751
 .000347601549309763
 .000446916277683981
 0
 0
 .00104280464792929
 .000148972092561327
 0
 .000645545734432416
 .000496573641871089
 0
 .000198629456748436
 4.96573641871089e-05
 4.96573641871089e-05
 0
 0
 0
 0
 0
 .000645545734432416
 .000546231006058199
 9.93147283742179e-05
 .000148972092561327
 0
 0
 0
 0
 .000347601549309763
 .000297944185122654
 0
 .000148972092561327
 9.93147283742179e-05
 .000148972092561327
 .000148972092561327
 0
 0
 0
 0
 0
 .000198629456748436
 .000198629456748436
 9.93147283742179e-05
 9.93147283742179e-05
 0
 0
 0
 0
 9.93147283742179e-05
 0
 0
 0
 0
 0
 .00129109146886483
 .000347601549309763
 .000347601549309763
 0
 0
 0
 0
 0
 0
 0
 0
 0
 0
 .00094348991955507
 .000893832555367961
 .000595888370245307
 0
 .000297944185122654
 .000297944185122654
 .000297944185122654
 0
 0
 0
 0
 0
 0
 0
 0
 0
 0
 0
 0
 0
 0
 0
 0
 0
 0
 0
 0
 0
 0
 0
 0
 0
 0
 0
 0
 0
 0
 4.96573641871089e-05
 4.96573641871089e-05
 4.96573641871089e-05
 4.96573641871089e-05
 4.96573641871089e-05
 4.96573641871089e-05
 4.96573641871089e-05
 0
 0
 .0033270434005363
 .00139040619723905
 .00134074883305194
 .000645545734432416
 .00193663720329725
 .00193663720329725
 .000297944185122654
 0
 0
 4.96573641871089e-05
 4.96573641871089e-05
 0
 0
 .0687754493991454
 .0360015890356539
 4.96573641871089e-05
 0
 .00148972092561327
 .000248286820935545
 0
 .000148972092561327
 9.93147283742179e-05
 4.96573641871089e-05
 0
 0
 0
 0
 0
 .000297944185122654
 .000297944185122654
 0
 0
 .00193663720329725
 .000595888370245307
 4.96573641871089e-05
 4.96573641871089e-05
 0
 0
 .000248286820935545
 0
 0
 .000248286820935545
 0
 0
 0
 4.96573641871089e-05
 0
 .000198629456748436
 4.96573641871089e-05
 0
 0
 0
 0
 0
 4.96573641871089e-05
 0
 0
 0
 0
 0
 .0167345317310557
 .0156420697189393
 .00402224649915583
 .00283046975866521
 .000148972092561327
 0
 0
 .000248286820935545
 .000248286820935545
 .000148972092561327
 .000347601549309763
 .000148972092561327
 9.93147283742179e-05
 .000645545734432416
 4.96573641871089e-05
 0
 0
 .000546231006058198
 0
 .000397258913496872
 0
 0
 0
 9.93147283742179e-05
 0
 0
 0
 0
 4.96573641871089e-05
 4.96573641871089e-05
 0
 0
 0
 0
 0
 0
 9.93147283742179e-05
 4.96573641871089e-05
 0
 0
 0
 0
 0
 0
 0
 0
 0
 0
 4.96573641871089e-05
 4.96573641871089e-05
 0
 0
 0
 0
 0
 0
 .000993147283742179
 .00094348991955507
 .000148972092561327
 0
 0
 .0131095441453968
 .0126626278677128
 .00799483563412454
 .00705134571456947
 .000446916277683981
 .000248286820935545
 0
 .000248286820935545
 0
 0
 9.93147283742179e-05
 9.93147283742179e-05
 0
 0
 0
 0
 9.93147283742179e-05
 0
 0
 0
 .0022345813884199
 .00119177674049061
 .000695203098619525
 .000297944185122654
 4.96573641871089e-05
 0
 0
 .000546231006058199
 4.96573641871089e-05
 4.96573641871089e-05
 0
 0
 0
 0
 0
 .000595888370245307
 .000148972092561327
 .000148972092561327
 4.96573641871089e-05
 .000297944185122654
 0
 0
 0
 0
 0
 0
 0
 0
 9.93147283742179e-05
 9.93147283742179e-05
 0
 4.96573641871089e-05
 4.96573641871089e-05
 .0174793921938624
 .000595888370245307
 .000148972092561327
 9.93147283742179e-05
 0
 0
 0
 .000397258913496872
 0
 0
 .0132585162379581
 .0124639984109644
 0
 0
 .000794517826993743
 0
 0
 0
 0
 .000148972092561327
 9.93147283742179e-05
 4.96573641871089e-05
 0
 0
 4.96573641871089e-05
 0
 .000297944185122654
 4.96573641871089e-05
 0
 0
 0
 4.96573641871089e-05
 0
 0
 .00208560929585858
 .0010924620121164
 .000198629456748436
 .000446916277683981
 .000148972092561327
 0
 4.96573641871089e-05
 0
 0
 .000794517826993743
 0
 0
 0
 0
 0
 .000148972092561327
 0
 .0145496077068229
 .000248286820935545
 .000148972092561327
 0
 4.96573641871089e-05
 4.96573641871089e-05
 .00158903565398749
 .0010924620121164
 0
 0
 0
 .000297944185122654
 .000198629456748436
 4.96573641871089e-05
 4.96573641871089e-05
 0
 4.96573641871089e-05
 0
 0
 0
 0
 0
 0
 0
 .00888866818949251
 .00273115503029099
 .0016386930181746
 .00144006356142616
 9.93147283742179e-05
 9.93147283742179e-05
 4.96573641871089e-05
 4.96573641871089e-05
 0
 0
 0
 .000546231006058198
 .000297944185122654
 0
 0
 .000198629456748436
 .000198629456748436
 0
 0
 .00590922633826597
 0
 0
 4.96573641871089e-05
 0
 0
 0
 4.96573641871089e-05
 4.96573641871089e-05
 0
 0
 0
 .000148972092561327
 0
 0
 0
 0
 0
 .000198629456748436
 .000148972092561327
 0
 0
 9.93147283742179e-05
 4.96573641871089e-05
 0
 0
 0
 0
 4.96573641871089e-05
 0
 0
 0
 4.96573641871089e-05
 0
 0
 .000695203098619525
 .000695203098619525
 0
 0
 9.93147283742179e-05
 0
 0
 0
 0
 4.96573641871089e-05
 4.96573641871089e-05
 4.96573641871089e-05
 0
 0
 0
 0
 0
 0
 0
 .000148972092561327
 4.96573641871089e-05
 0
 0
 0
 0
 0
 .000595888370245307
 0
 .000347601549309763
 4.96573641871089e-05
 0
 .000248286820935545
 0
 0
 0
 0
 .00198629456748436
 .0010924620121164
 0
 4.96573641871089e-05
 0
 0
 .00213526666004568
 .00213526666004568
 .00208560929585858
 .000844175191180852
 .000446916277683981
 0
 .000148972092561327
 0
 0
 0
 0
 0
 0
 0
 0
 0
 0
 0
 0
 0
 0
 0
 0
 .00392293177078161
 .00342635812891052
 .000595888370245307
 .000148972092561327
 0
 0
 .00283046975866521
 .00134074883305194
 .000198629456748436
 .000148972092561327
 0
 0
 .000496573641871089
 .000397258913496872
 .000297944185122654
 .000198629456748436
 9.93147283742179e-05
 0
 0
 .000347601549309763
 .000347601549309763
 .000148972092561327
 0
 0
 0
 .0240341642665607
 .0141026914291389
 .0141026914291389
 .00516436587545933
 .00292978448703943
 .0010924620121164
 .000993147283742179
 .000297944185122654
 .000148972092561327
 0
 0
 .00993147283742179
 4.96573641871089e-05
 4.96573641871089e-05
 4.96573641871089e-05
 0
 0
 4.96573641871089e-05
 4.96573641871089e-05
 0
 0
 .00148972092561327
 4.96573641871089e-05
 .00839209454762141
 0
 .00824312245506009
 0
 0
