# Supplementary material for: Fermentation couples Chloroflexi and sulfate-reducing bacteria to Cyanobacteria in hypersaline microbial mats
Source: Front Microbiol. 2014 Feb 26;5:61. doi: 10.3389/fmicb.2014.00061 (PMC3935151; doi:10.3389/fmicb.2014.00061)
Supplement: Supplementary file 1 [file Krona_charts_supplemental.zip › OTU table krona/GNI_LY_1200_DNA_otutable.html]

Javascript must be enabled to view this page.

magnitude
 .999999999999953
 .994276290259945
 .0013581684128832
 .00106713232440823
 .00106713232440823
 0
 0
 0
 .000291036088474971
 .000291036088474971
 9.7012029491657e-05
 .00324990298797051
 .00324990298797051
 9.7012029491657e-05
 9.7012029491657e-05
 9.7012029491657e-05
 4.85060147458285e-05
 .001697710516104
 .00111563833915406
 .00097012029491657
 0
 0
 4.85060147458285e-05
 9.7012029491657e-05
 4.85060147458285e-05
 0
 0
 0
 9.7012029491657e-05
 9.7012029491657e-05
 .00116414435389988
 .000242530073729142
 4.85060147458285e-05
 0
 0
 0
 0
 .106325184322856
 .000194024058983314
 .000194024058983314
 9.7012029491657e-05
 .000679084206441599
 .000679084206441599
 4.85060147458285e-05
 0
 0
 .0551998447807527
 .0547147846332944
 .026872332169189
 .00388048117966628
 .00223127667830811
 0
 4.85060147458285e-05
 0
 0
 0
 .00310438494373302
 0
 0
 0
 0
 .0326445479239426
 .0325960419091968
 .0231373690337602
 .000194024058983314
 .000194024058983314
 .000921614280170741
 0
 .000194024058983314
 .000291036088474971
 0
 4.85060147458285e-05
 4.85060147458285e-05
 4.85060147458285e-05
 0
 .00106713232440823
 0
 0
 .0176076833527357
 .0174621653084982
 .00882809468374078
 .000533566162204113
 .000145518044237485
 4.85060147458285e-05
 .00266783081102057
 4.85060147458285e-05
 0
 0
 0
 .00063057819169577
 .00392898719441211
 .00392898719441211
 .00344392704695382
 4.85060147458285e-05
 0
 4.85060147458285e-05
 4.85060147458285e-05
 4.85060147458285e-05
 0
 .00111563833915406
 0
 0
 .00111563833915406
 .00111563833915406
 .000776096235933256
 .384458672875429
 .192617384555684
 .191550252231275
 .133731082654249
 .023767947225456
 .00955568490492821
 4.85060147458285e-05
 .0133391540551028
 .0126600698486612
 .00727590221187426
 0
 .178162592161428
 .169334497477687
 .0178502134264649
 .000339542103220799
 .118112145906093
 .0465657741559953
 .0327415599534343
 .00829452852153667
 .00620876988746604
 .00509313154831199
 .00373496313542879
 .000339542103220799
 .000339542103220799
 .000291036088474971
 .182382615444314
 .182334109429569
 .00324990298797051
 .00295886689949554
 4.85060147458285e-05
 0
 0
 0
 .00145518044237485
 0
 0
 9.7012029491657e-05
 4.85060147458285e-05
 0
 0
 0
 0
 4.85060147458285e-05
 0
 0
 0
 0
 0
 .000242530073729142
 .000145518044237485
 0
 0
 0
 0
 0
 0
 .176998447807528
 .172244858362437
 .000436554132712456
 0
 4.85060147458285e-05
 .105403570042686
 .0790648040357004
 .00179472254559565
 0
 .00756693830034924
 .00417151726814125
 9.7012029491657e-05
 0
 0
 0
 0
 0
 0
 .0314804035700427
 .000824602250679084
 .000194024058983314
 0
 0
 4.85060147458285e-05
 4.85060147458285e-05
 0
 .000145518044237485
 9.7012029491657e-05
 0
 .000194024058983314
 9.7012029491657e-05
 0
 0
 0
 0
 4.85060147458285e-05
 4.85060147458285e-05
 .000776096235933256
 .000485060147458285
 0
 4.85060147458285e-05
 .000194024058983314
 9.7012029491657e-05
 4.85060147458285e-05
 0
 .000436554132712456
 .000194024058983314
 0
 4.85060147458285e-05
 4.85060147458285e-05
 4.85060147458285e-05
 4.85060147458285e-05
 4.85060147458285e-05
 4.85060147458285e-05
 4.85060147458285e-05
 0
 0
 .0027163368257664
 .0027163368257664
 .00266783081102057
 .00160069848661234
 4.85060147458285e-05
 0
 .000727590221187427
 9.7012029491657e-05
 0
 0
 0
 0
 0
 0
 .00354093907644548
 .000776096235933256
 .000679084206441599
 .000194024058983314
 0
 0
 .000145518044237485
 9.7012029491657e-05
 4.85060147458285e-05
 0
 0
 0
 0
 .00276484284051222
 .00194024058983314
 .00106713232440823
 0
 0
 0
 0
 .000194024058983314
 .000194024058983314
 .000145518044237485
 .000145518044237485
 0
 0
 0
 0
 0
 0
 4.85060147458285e-05
 4.85060147458285e-05
 .000485060147458285
 .000485060147458285
 .000485060147458285
 .000339542103220799
 .000194024058983314
 .000145518044237485
 .000145518044237485
 9.7012029491657e-05
 9.7012029491657e-05
 4.85060147458285e-05
 4.85060147458285e-05
 .000194024058983314
 .000194024058983314
 .000194024058983314
 0
 0
 0
 0
 .00111563833915406
 .00111563833915406
 .00111563833915406
 .00106713232440823
 .00106713232440823
 .0010186263096624
 .000436554132712456
 .000291036088474971
 0
 .0284245246410555
 .0109623593325572
 .0109623593325572
 .00291036088474971
 .0174621653084982
 .0173166472642608
 .00281334885525805
 9.7012029491657e-05
 9.7012029491657e-05
 4.85060147458285e-05
 4.85060147458285e-05
 .000145518044237485
 4.85060147458285e-05
 .223903764066743
 .10918703919286
 .000194024058983314
 0
 .000436554132712456
 .000291036088474971
 0
 .000291036088474971
 .000194024058983314
 0
 0
 9.7012029491657e-05
 9.7012029491657e-05
 0
 0
 .00339542103220799
 .00310438494373302
 .00174621653084983
 .00160069848661234
 .00412301125339542
 .00097012029491657
 0
 0
 0
 0
 .000388048117966628
 0
 0
 .00121265036864571
 0
 0
 0
 .000242530073729142
 9.7012029491657e-05
 .000194024058983314
 0
 0
 4.85060147458285e-05
 0
 .000145518044237485
 0
 4.85060147458285e-05
 0
 0
 0
 0
 0
 .0454501358168412
 .042103220799379
 .0108653473030656
 .00737291424136593
 .00097012029491657
 0
 .000485060147458285
 .00378346915017462
 .00179472254559565
 .000582072176949942
 .000388048117966628
 .00616026387272022
 .00577221575475359
 .00164920450135817
 .000679084206441599
 9.7012029491657e-05
 4.85060147458285e-05
 .00164920450135817
 0
 .0013581684128832
 .000485060147458285
 .000485060147458285
 4.85060147458285e-05
 .000339542103220799
 0
 0
 4.85060147458285e-05
 0
 .000921614280170741
 .000727590221187427
 9.7012029491657e-05
 9.7012029491657e-05
 0
 9.7012029491657e-05
 9.7012029491657e-05
 0
 4.85060147458285e-05
 4.85060147458285e-05
 4.85060147458285e-05
 4.85060147458285e-05
 0
 .000145518044237485
 9.7012029491657e-05
 4.85060147458285e-05
 4.85060147458285e-05
 4.85060147458285e-05
 4.85060147458285e-05
 4.85060147458285e-05
 0
 0
 0
 0
 0
 0
 0
 0
 .00194024058983314
 .00160069848661234
 .000242530073729142
 4.85060147458285e-05
 0
 .0289580908032597
 .027163368257664
 .0176561893674816
 .0149398525417152
 .00097012029491657
 .00184322856034148
 0
 .00140667442762903
 0
 0
 0
 0
 0
 0
 0
 4.85060147458285e-05
 .000145518044237485
 0
 0
 0
 .0260477299185099
 .0226523088863019
 .0181897555296857
 .00926464881645324
 .00227978269305394
 0
 4.85060147458285e-05
 .00242530073729142
 .000921614280170741
 .000921614280170741
 4.85060147458285e-05
 0
 0
 4.85060147458285e-05
 4.85060147458285e-05
 .0027163368257664
 0
 0
 0
 .00247380675203725
 0
 0
 0
 0
 0
 0
 0
 0
 .000242530073729142
 .000194024058983314
 0
 0
 0
 .039871944121071
 .00334691501746217
 .00189173457508731
 .00106713232440823
 .000145518044237485
 9.7012029491657e-05
 0
 .000533566162204113
 0
 0
 .0198874660457897
 .0174136592937524
 .000242530073729142
 4.85060147458285e-05
 .00276484284051222
 0
 0
 0
 4.85060147458285e-05
 .000242530073729142
 4.85060147458285e-05
 .000145518044237485
 0
 0
 4.85060147458285e-05
 0
 .00509313154831199
 4.85060147458285e-05
 0
 0
 4.85060147458285e-05
 4.85060147458285e-05
 0
 0
 .00538416763678696
 .001697710516104
 .000194024058983314
 .00286185487000388
 .000533566162204113
 4.85060147458285e-05
 0
 0
 0
 .00261932479627474
 9.7012029491657e-05
 4.85060147458285e-05
 4.85060147458285e-05
 0
 4.85060147458285e-05
 0
 0
 .0720799379123012
 .00121265036864571
 .000824602250679084
 4.85060147458285e-05
 0
 0
 .0159584788513776
 .00877958866899494
 .000242530073729142
 0
 0
 .00121265036864571
 .000679084206441599
 .000485060147458285
 .000194024058983314
 4.85060147458285e-05
 0
 4.85060147458285e-05
 4.85060147458285e-05
 .00150368645712068
 .00121265036864571
 .000824602250679084
 0
 9.7012029491657e-05
 .0397749320915794
 .014697322467986
 .0052871556072953
 .00460807140085371
 .000291036088474971
 9.7012029491657e-05
 0
 0
 4.85060147458285e-05
 4.85060147458285e-05
 0
 .00470508343034536
 .00174621653084983
 .000194024058983314
 0
 4.85060147458285e-05
 0
 9.7012029491657e-05
 4.85060147458285e-05
 .0202755141637563
 9.7012029491657e-05
 4.85060147458285e-05
 .00130966239813737
 0
 0
 4.85060147458285e-05
 .000485060147458285
 4.85060147458285e-05
 0
 0
 .000145518044237485
 .00150368645712068
 .000339542103220799
 .000242530073729142
 .000436554132712456
 .000291036088474971
 0
 .000921614280170741
 .000921614280170741
 4.85060147458285e-05
 4.85060147458285e-05
 .000291036088474971
 .000194024058983314
 0
 0
 0
 0
 0
 0
 0
 0
 0
 0
 0
 .00266783081102057
 .00247380675203725
 0
 0
 .00126115638339154
 .000485060147458285
 .000145518044237485
 0
 9.7012029491657e-05
 .000339542103220799
 .000242530073729142
 .000194024058983314
 4.85060147458285e-05
 0
 0
 0
 0
 0
 0
 .00266783081102057
 .000679084206441599
 0
 0
 .000339542103220799
 .000339542103220799
 4.85060147458285e-05
 .00184322856034148
 0
 .00116414435389988
 .000194024058983314
 0
 .000388048117966628
 .000242530073729142
 .000242530073729142
 4.85060147458285e-05
 4.85060147458285e-05
 .00397749320915794
 .00140667442762903
 0
 .000242530073729142
 4.85060147458285e-05
 4.85060147458285e-05
 .0258051998447808
 .0258051998447808
 .0257081878152891
 .0223612727978269
 .00989522700814901
 0
 .00291036088474971
 0
 9.7012029491657e-05
 0
 0
 9.7012029491657e-05
 9.7012029491657e-05
 4.85060147458285e-05
 0
 0
 9.7012029491657e-05
 9.7012029491657e-05
 0
 9.7012029491657e-05
 9.7012029491657e-05
 9.7012029491657e-05
 4.85060147458285e-05
 .0203240201785021
 .0162495149398525
 .00320139697322468
 .00063057819169577
 0
 0
 .0129025999223904
 .00931315483119906
 .0030558789289872
 .00208575863407063
 .000242530073729142
 .000242530073729142
 .00383197516492045
 .00378346915017462
 .00310438494373302
 .00227978269305394
 .00194024058983314
 0
 4.85060147458285e-05
 .00397749320915794
 .00397749320915794
 .00164920450135817
 .000194024058983314
 0
 0
 .00485060147458285
 .00276484284051222
 .00276484284051222
 .00063057819169577
 .001697710516104
 .000824602250679084
 .000824602250679084
 .000436554132712456
 0
 0
 0
 .00208575863407062
 .000291036088474971
 .000291036088474971
 .000291036088474971
 4.85060147458285e-05
 4.85060147458285e-05
 .000194024058983314
 .000194024058983314
 4.85060147458285e-05
 4.85060147458285e-05
 0
 0
 .00179472254559565
 0
 .00179472254559565
 0
 0
