# Supplementary material for: Fermentation couples Chloroflexi and sulfate-reducing bacteria to Cyanobacteria in hypersaline microbial mats
Source: Front Microbiol. 2014 Feb 26;5:61. doi: 10.3389/fmicb.2014.00061 (PMC3935151; doi:10.3389/fmicb.2014.00061)
Supplement: Supplementary file 1 [file Krona_charts_supplemental.zip › OTU table krona/GNI_LY_2400_cDNA_otutable.html]

Javascript must be enabled to view this page.

magnitude
 1.00000000000002
 .93810123168212
 .000539422817585184
 .000314663310258024
 .000314663310258024
 0
 0
 0
 .00022475950732716
 4.4951901465432e-05
 0
 .00296682549671851
 .00296682549671851
 0
 0
 0
 0
 .00215769127034074
 .00184302796008271
 .00179807605861728
 8.9903802930864e-05
 .000134855704396296
 4.4951901465432e-05
 0
 0
 4.4951901465432e-05
 0
 0
 0
 0
 .000809134226377776
 .000179807605861728
 0
 0
 0
 0
 0
 .0322754652521801
 .00103389373370494
 .00103389373370494
 4.4951901465432e-05
 .000314663310258024
 .000314663310258024
 0
 .000179807605861728
 0
 .0178908567832419
 .0178908567832419
 .00768677515058888
 .000179807605861728
 4.4951901465432e-05
 0
 0
 0
 0
 0
 .0021127393688753
 4.4951901465432e-05
 4.4951901465432e-05
 0
 0
 .00777667895351974
 .00777667895351974
 .00557403578171357
 .000494470916119752
 .00044951901465432
 .000314663310258024
 .00022475950732716
 0
 .00022475950732716
 8.9903802930864e-05
 8.9903802930864e-05
 4.4951901465432e-05
 0
 0
 .000134855704396296
 0
 0
 .00525937247145555
 .00525937247145555
 .00382091162456172
 .000314663310258024
 8.9903802930864e-05
 0
 .000179807605861728
 0
 8.9903802930864e-05
 0
 0
 .00022475950732716
 0
 0
 0
 0
 0
 0
 0
 0
 0
 .000179807605861728
 0
 0
 .000179807605861728
 .000179807605861728
 .000179807605861728
 .0512002157691269
 .00867571698282838
 .00867571698282838
 .00485480535826666
 .000539422817585184
 .000134855704396296
 0
 .00193293176301357
 .00193293176301357
 .0011237975366358
 0
 .040591567023285
 .0381641643441517
 .00301177739818394
 0
 .0276903713027061
 .0107884563517037
 .00710240043153826
 .0023824507776679
 .0021127393688753
 .00193293176301358
 .00166322035422098
 0
 0
 0
 .751460936797634
 .751460936797634
 .00485480535826666
 .0044951901465432
 8.9903802930864e-05
 4.4951901465432e-05
 0
 0
 .00134855704396296
 0
 0
 .00089903802930864
 0
 0
 0
 .000179807605861728
 .000134855704396296
 0
 0
 .000179807605861728
 .000179807605861728
 0
 0
 .00022475950732716
 .000134855704396296
 4.4951901465432e-05
 0
 0
 0
 0
 0
 .745257574395404
 .742965027420666
 .00346129641283826
 0
 0
 .645419401240675
 .534073541310798
 .0101141778297222
 0
 .0344781084239863
 .0161377326260901
 .00107884563517037
 .000494470916119752
 4.4951901465432e-05
 0
 0
 0
 0
 .0147442236806617
 .000179807605861728
 .000179807605861728
 0
 4.4951901465432e-05
 .00130360514249753
 .00044951901465432
 4.4951901465432e-05
 .000314663310258024
 .000314663310258024
 0
 0
 0
 4.4951901465432e-05
 4.4951901465432e-05
 0
 0
 0
 0
 .000359615211723456
 .000359615211723456
 0
 8.9903802930864e-05
 4.4951901465432e-05
 0
 0
 4.4951901465432e-05
 .00044951901465432
 .000404567113188888
 0
 .000134855704396296
 .000134855704396296
 .000134855704396296
 .000134855704396296
 0
 0
 0
 0
 0
 4.4951901465432e-05
 4.4951901465432e-05
 4.4951901465432e-05
 4.4951901465432e-05
 0
 0
 0
 0
 0
 0
 0
 0
 0
 0
 .00274206598939135
 .000179807605861728
 8.9903802930864e-05
 0
 0
 0
 0
 0
 0
 0
 8.9903802930864e-05
 0
 0
 .00256225838352962
 .0022475950732716
 .00089903802930864
 0
 .000943989930774072
 .000943989930774072
 .000943989930774072
 .00022475950732716
 .000134855704396296
 4.4951901465432e-05
 4.4951901465432e-05
 8.9903802930864e-05
 0
 0
 0
 0
 0
 0
 0
 .00022475950732716
 .00022475950732716
 .00022475950732716
 .000134855704396296
 0
 0
 0
 0
 0
 0
 0
 0
 0
 0
 0
 0
 0
 0
 4.4951901465432e-05
 4.4951901465432e-05
 4.4951901465432e-05
 4.4951901465432e-05
 4.4951901465432e-05
 4.4951901465432e-05
 0
 0
 0
 .00791153465791604
 .0022475950732716
 .0022475950732716
 .000943989930774072
 .00566393958464443
 .00552908388024814
 .000269711408792592
 4.4951901465432e-05
 4.4951901465432e-05
 0
 0
 0
 0
 .0815427492582933
 .0338937337049357
 0
 0
 .00148341274835926
 .00044951901465432
 0
 .00044951901465432
 .000359615211723456
 0
 0
 0
 0
 4.4951901465432e-05
 4.4951901465432e-05
 8.9903802930864e-05
 8.9903802930864e-05
 8.9903802930864e-05
 8.9903802930864e-05
 .000988941832239504
 .00022475950732716
 0
 0
 4.4951901465432e-05
 4.4951901465432e-05
 .000134855704396296
 0
 0
 8.9903802930864e-05
 0
 0
 0
 .000134855704396296
 0
 0
 0
 0
 0
 0
 0
 0
 0
 0
 0
 4.4951901465432e-05
 0
 0
 .0067427852198148
 .0058886990919716
 .000719230423446912
 .000404567113188888
 0
 0
 0
 4.4951901465432e-05
 0
 0
 8.9903802930864e-05
 8.9903802930864e-05
 8.9903802930864e-05
 .000179807605861728
 0
 8.9903802930864e-05
 0
 .000269711408792592
 0
 .000269711408792592
 0
 0
 4.4951901465432e-05
 0
 0
 0
 0
 4.4951901465432e-05
 0
 0
 0
 0
 0
 4.4951901465432e-05
 4.4951901465432e-05
 0
 0
 0
 0
 0
 0
 0
 0
 0
 0
 0
 0
 0
 0
 0
 0
 0
 0
 0
 0
 0
 .000584374719050616
 .000539422817585184
 .000134855704396296
 0
 0
 .023105277353232
 .0225209026341814
 .0157781174143666
 .0130810033264407
 .00166322035422098
 .000764182324912344
 0
 .000584374719050616
 0
 0
 0
 0
 0
 0
 0
 0
 .000494470916119752
 .000494470916119752
 0
 0
 .000943989930774072
 .00067427852198148
 .00044951901465432
 .00022475950732716
 8.9903802930864e-05
 0
 0
 .00022475950732716
 0
 0
 0
 0
 0
 0
 0
 .00269711408792592
 0
 0
 0
 .00116874943810123
 0
 0
 0
 .000269711408792592
 0
 0
 0
 0
 .00148341274835926
 .00139350894542839
 .000404567113188888
 0
 0
 .0249483053133147
 .00044951901465432
 .000134855704396296
 0
 0
 0
 0
 .000134855704396296
 4.4951901465432e-05
 0
 .0189697024184123
 .0170367706553987
 0
 0
 .00103389373370494
 0
 0
 8.9903802930864e-05
 4.4951901465432e-05
 0
 0
 0
 0
 0
 .000584374719050616
 0
 .000854086127843208
 .000359615211723456
 .000314663310258024
 8.9903802930864e-05
 0
 .000134855704396296
 8.9903802930864e-05
 4.4951901465432e-05
 .00359615211723456
 .00152836464982469
 .00022475950732716
 .000494470916119752
 .00022475950732716
 0
 .000179807605861728
 0
 0
 .00044951901465432
 0
 0
 0
 0
 0
 4.4951901465432e-05
 0
 .0199586442506518
 .000404567113188888
 .000404567113188888
 0
 .000179807605861728
 .000179807605861728
 .00220264317180617
 .00202283556594444
 8.9903802930864e-05
 0
 0
 .000764182324912344
 .000134855704396296
 0
 0
 0
 0
 0
 0
 0
 0
 0
 0
 0
 .0134406185381642
 .00638317000809135
 .00418052683628518
 .0035062483143037
 .000494470916119752
 .000134855704396296
 0
 0
 0
 0
 0
 .000494470916119752
 .000314663310258024
 4.4951901465432e-05
 0
 8.9903802930864e-05
 8.9903802930864e-05
 4.4951901465432e-05
 0
 .0067427852198148
 0
 0
 8.9903802930864e-05
 .00022475950732716
 .00022475950732716
 0
 4.4951901465432e-05
 4.4951901465432e-05
 0
 0
 0
 .000134855704396296
 0
 0
 4.4951901465432e-05
 4.4951901465432e-05
 0
 .000179807605861728
 8.9903802930864e-05
 0
 0
 0
 0
 0
 0
 0
 0
 4.4951901465432e-05
 4.4951901465432e-05
 4.4951901465432e-05
 4.4951901465432e-05
 0
 0
 0
 .00067427852198148
 .000539422817585184
 0
 0
 8.9903802930864e-05
 0
 0
 0
 0
 8.9903802930864e-05
 0
 0
 0
 0
 0
 0
 0
 0
 0
 .000134855704396296
 4.4951901465432e-05
 4.4951901465432e-05
 4.4951901465432e-05
 0
 0
 0
 .000494470916119752
 0
 .000359615211723456
 8.9903802930864e-05
 0
 0
 0
 0
 0
 0
 .00193293176301357
 .00103389373370494
 0
 .000179807605861728
 0
 0
 .00251730648206419
 .00251730648206419
 .00251730648206419
 .00134855704396296
 .00022475950732716
 0
 8.9903802930864e-05
 0
 0
 0
 0
 0
 0
 0
 0
 0
 0
 0
 0
 0
 0
 0
 0
 .00364110401869999
 .00296682549671851
 .00067427852198148
 .00022475950732716
 0
 0
 .00229254697473703
 .00148341274835926
 .000134855704396296
 8.9903802930864e-05
 0
 0
 .00067427852198148
 .00067427852198148
 .00022475950732716
 .000134855704396296
 4.4951901465432e-05
 0
 0
 .000809134226377776
 .000809134226377776
 .000584374719050616
 4.4951901465432e-05
 0
 0
 .0527285804189517
 .0372201744133777
 .0372201744133777
 .00943989930774071
 .0156882136114358
 .00602355479636789
 .00597860289490246
 .00238245077766789
 4.4951901465432e-05
 0
 4.4951901465432e-05
 .015508406005574
 .000134855704396296
 .000134855704396296
 .000134855704396296
 .000134855704396296
 .000134855704396296
 0
 0
 0
 0
 .00364110401869999
 .000539422817585184
 .0116874943810123
 4.4951901465432e-05
 .0112829272678234
 4.4951901465432e-05
 0
