# Supplementary material for: Fermentation couples Chloroflexi and sulfate-reducing bacteria to Cyanobacteria in hypersaline microbial mats
Source: Front Microbiol. 2014 Feb 26;5:61. doi: 10.3389/fmicb.2014.00061 (PMC3935151; doi:10.3389/fmicb.2014.00061)
Supplement: Supplementary file 1 [file Krona_charts_supplemental.zip › OTU table krona/GNI_LY_2400_DNA_otutable.html]

Javascript must be enabled to view this page.

magnitude
 .999999999999956
 .996392682298333
 .00141716052563772
 .00122391136305076
 .00122391136305076
 0
 0
 0
 .000193249162586962
 .000193249162586962
 0
 .00418706518938418
 .00418706518938418
 .000322081937644937
 .000322081937644937
 .000322081937644937
 .000193249162586962
 .0020613244009276
 .00135274413810873
 .00115949497552177
 0
 0
 0
 0
 0
 6.44163875289874e-05
 6.44163875289874e-05
 0
 .000322081937644937
 .000322081937644937
 .000966245812934811
 6.44163875289874e-05
 0
 6.44163875289874e-05
 0
 0
 0
 .114467920639011
 .000128832775057975
 .000128832775057975
 0
 .000708580262818861
 .000579747487760886
 0
 .000128832775057975
 0
 .0615176500901827
 .0613888173151247
 .0323370265395516
 .00521772738984798
 .00283432105127544
 0
 0
 0
 0
 0
 .00392939963926823
 0
 0
 0
 0
 .0309842824014429
 .0308554496263849
 .0231254831229064
 .00025766555011595
 .00025766555011595
 .00225457356351456
 6.44163875289874e-05
 .000193249162586962
 .000193249162586962
 0
 0
 0
 0
 6.44163875289874e-05
 .0010306622004638
 .000128832775057975
 .000128832775057975
 .0211285751095078
 .0207420767843339
 .00940479257923216
 .000772996650347848
 .000322081937644937
 0
 .00289873743880443
 0
 .000128832775057975
 0
 .000128832775057975
 .00199690801339861
 .00251223911363051
 .00251223911363051
 .00219015717598557
 .00025766555011595
 6.44163875289874e-05
 0
 0
 0
 0
 .000515331100231899
 0
 0
 .000515331100231899
 .000515331100231899
 .000322081937644937
 .212187580520486
 .0856737954135536
 .0849652151507347
 .0526281886111825
 .00521772738984798
 .00141716052563772
 0
 .0117237825302757
 .0115305333676887
 .00760113372842051
 0
 .114790002576656
 .109572275186808
 .010306622004638
 .000128832775057975
 .0771708322597267
 .0309198660139139
 .0198402473589281
 .00502447822726102
 .00431589796444215
 .00322081937644937
 .00186807523834063
 0
 0
 0
 .165034784849267
 .165034784849267
 .00296315382633342
 .00264107188868848
 0
 0
 0
 0
 .000128832775057975
 0
 0
 .00025766555011595
 6.44163875289874e-05
 0
 0
 6.44163875289874e-05
 6.44163875289874e-05
 0
 0
 0
 0
 0
 0
 6.44163875289874e-05
 0
 0
 0
 0
 0
 0
 0
 .159623808296832
 .154728162844628
 .000901829425405823
 0
 0
 .112277763463025
 .0914712702911621
 .00199690801339861
 0
 .0056042257150219
 .00244782272610152
 0
 .000193249162586962
 0
 0
 0
 0
 0
 .00772996650347849
 .000450914712702912
 .000128832775057975
 0
 0
 .000128832775057975
 0
 0
 6.44163875289874e-05
 6.44163875289874e-05
 0
 6.44163875289874e-05
 6.44163875289874e-05
 0
 0
 .000128832775057975
 .000128832775057975
 0
 0
 .00167482607575367
 .00115949497552177
 0
 0
 .000193249162586962
 6.44163875289874e-05
 6.44163875289874e-05
 0
 .000193249162586962
 6.44163875289874e-05
 0
 0
 0
 6.44163875289874e-05
 6.44163875289874e-05
 0
 0
 0
 0
 0
 .0056042257150219
 .0056042257150219
 .00541097655243494
 .00328523576397836
 0
 0
 .00115949497552177
 0
 .000128832775057975
 0
 0
 0
 0
 0
 .00231898995104355
 .000579747487760886
 .000450914712702912
 6.44163875289874e-05
 0
 0
 0
 0
 0
 0
 0
 0
 0
 .00173924246328266
 .00128832775057975
 .000772996650347849
 6.44163875289874e-05
 0
 0
 0
 .000193249162586962
 6.44163875289874e-05
 0
 0
 6.44163875289874e-05
 6.44163875289874e-05
 6.44163875289874e-05
 0
 .000128832775057975
 6.44163875289874e-05
 0
 0
 .00141716052563772
 .00141716052563772
 .00128832775057975
 .00109507858799279
 .00025766555011595
 0
 0
 0
 0
 0
 0
 0
 0
 0
 0
 0
 0
 0
 .000193249162586962
 .000193249162586962
 .000193249162586962
 .000193249162586962
 .000193249162586962
 .000193249162586962
 .000128832775057975
 0
 0
 .0462509662458127
 .0135918577686163
 .0135918577686163
 .00541097655243494
 .0326591084771965
 .0324658593146095
 .00450914712702912
 .00025766555011595
 .00025766555011595
 6.44163875289874e-05
 0
 .000386498325173924
 0
 .376191703169283
 .181138881731514
 .000322081937644937
 .00025766555011595
 .0015459933006957
 .000901829425405823
 6.44163875289874e-05
 .000644163875289874
 .000386498325173924
 .000128832775057975
 6.44163875289874e-05
 6.44163875289874e-05
 6.44163875289874e-05
 .00025766555011595
 .00025766555011595
 .00244782272610152
 .00244782272610152
 .00135274413810873
 .00135274413810873
 .00830971399123938
 .0015459933006957
 .00025766555011595
 .00025766555011595
 0
 0
 .00141716052563772
 0
 0
 .00180365885081165
 .000128832775057975
 6.44163875289874e-05
 6.44163875289874e-05
 0
 0
 .000128832775057975
 .00025766555011595
 0
 6.44163875289874e-05
 0
 .00025766555011595
 0
 .000128832775057975
 6.44163875289874e-05
 0
 0
 0
 0
 .0451558876578199
 .0406467405307909
 .00753671734089153
 .00534656016490595
 .00180365885081165
 0
 .000644163875289874
 .00289873743880443
 .0015459933006957
 .000644163875289874
 .000837413037876836
 .0066348879154857
 .0061195568152538
 .00148157691316671
 .000515331100231899
 .000128832775057975
 0
 .00199690801339861
 .000193249162586962
 .00161040968822468
 .000322081937644937
 .000193249162586962
 .000193249162586962
 .00025766555011595
 0
 .000128832775057975
 0
 6.44163875289874e-05
 .000644163875289874
 .000515331100231899
 0
 .000128832775057975
 6.44163875289874e-05
 .000128832775057975
 6.44163875289874e-05
 6.44163875289874e-05
 .000644163875289874
 .000386498325173924
 .000193249162586962
 6.44163875289874e-05
 0
 0
 0
 0
 6.44163875289874e-05
 0
 0
 0
 0
 0
 6.44163875289874e-05
 0
 6.44163875289874e-05
 6.44163875289874e-05
 0
 0
 .00238340633857253
 .00141716052563772
 .000322081937644937
 6.44163875289874e-05
 0
 .0949497552177277
 .0902473589281114
 .0568152538005668
 .0441252254573563
 .00669930430301469
 .00669930430301469
 .000837413037876836
 .00418706518938418
 .000515331100231899
 0
 0
 0
 6.44163875289874e-05
 6.44163875289874e-05
 6.44163875289874e-05
 0
 .000193249162586962
 6.44163875289874e-05
 .000128832775057975
 6.44163875289874e-05
 .0271837155372326
 .0220948209224427
 .0169415099201237
 .00740788456583355
 .00334965215150734
 6.44163875289874e-05
 0
 .00354290131409431
 .000644163875289874
 .000450914712702912
 6.44163875289874e-05
 0
 0
 6.44163875289874e-05
 0
 .00863179592888431
 0
 0
 0
 .00605514042772482
 0
 0
 0
 .000837413037876836
 6.44163875289874e-05
 0
 0
 0
 .00219015717598557
 .00212574078845658
 6.44163875289874e-05
 6.44163875289874e-05
 6.44163875289874e-05
 .0378768358670444
 .00289873743880443
 .00199690801339861
 .0010306622004638
 0
 .000450914712702912
 6.44163875289874e-05
 .000450914712702912
 0
 0
 .0123035300180366
 .010048956454522
 0
 0
 .00231898995104355
 6.44163875289874e-05
 6.44163875289874e-05
 0
 0
 .000772996650347848
 0
 .000772996650347848
 0
 0
 .000708580262818861
 0
 .00676372069054368
 .000515331100231899
 .000450914712702912
 .000128832775057975
 0
 .000579747487760886
 6.44163875289874e-05
 6.44163875289874e-05
 .00850296315382634
 .00322081937644937
 .000579747487760886
 .00322081937644937
 .0010306622004638
 .000193249162586962
 .000128832775057975
 0
 6.44163875289874e-05
 .00283432105127545
 6.44163875289874e-05
 0
 0
 0
 6.44163875289874e-05
 0
 0
 .148544189641846
 .00148157691316671
 .000772996650347849
 0
 .000450914712702912
 .000386498325173924
 .0330456068023704
 .0232543158979644
 .000322081937644937
 0
 0
 .00328523576397836
 .00109507858799279
 .000579747487760886
 .000128832775057975
 0
 6.44163875289874e-05
 .000193249162586962
 0
 .000837413037876836
 .000579747487760886
 .000193249162586962
 .000128832775057975
 0
 .0837413037876837
 .0439963926822983
 .0145581035815511
 .0123679464055656
 .00161040968822468
 .000644163875289874
 .00025766555011595
 .000128832775057975
 .000193249162586962
 0
 0
 .0132053594434424
 .00470239628961608
 6.44163875289874e-05
 .000128832775057975
 .000193249162586962
 .000128832775057975
 .000128832775057975
 0
 .0347204328781241
 .000128832775057975
 6.44163875289874e-05
 .00167482607575367
 .000450914712702912
 .000386498325173924
 0
 .00122391136305076
 .000128832775057975
 6.44163875289874e-05
 .000386498325173924
 .000193249162586962
 .000966245812934811
 .00025766555011595
 .000128832775057975
 .00025766555011595
 .000193249162586962
 0
 .00244782272610152
 .00186807523834063
 .000128832775057975
 .000128832775057975
 .000322081937644937
 .000128832775057975
 .00025766555011595
 .00025766555011595
 6.44163875289874e-05
 .000128832775057975
 .000128832775057975
 0
 0
 0
 .000128832775057975
 6.44163875289874e-05
 0
 .0106931203298119
 .010113372842051
 0
 0
 .00186807523834063
 .00115949497552177
 .000128832775057975
 0
 0
 .000386498325173924
 .000128832775057975
 .000128832775057975
 0
 0
 0
 0
 6.44163875289874e-05
 .000128832775057975
 .000128832775057975
 .00566864210255089
 .00186807523834063
 6.44163875289874e-05
 6.44163875289874e-05
 .000128832775057975
 6.44163875289874e-05
 0
 .0015459933006957
 0
 .000515331100231899
 0
 0
 .000644163875289874
 .000386498325173924
 .000386498325173924
 .00025766555011595
 .000193249162586962
 .0056042257150219
 .00199690801339861
 0
 .000193249162586962
 0
 0
 .0314996135016747
 .0314996135016747
 .0313707807266168
 .0276990466374645
 .00637722236536975
 6.44163875289874e-05
 .00238340633857253
 0
 .000193249162586962
 6.44163875289874e-05
 0
 0
 0
 0
 0
 0
 0
 0
 0
 0
 0
 0
 0
 .0289229580005153
 .0224813192476166
 .0056042257150219
 .00251223911363051
 0
 0
 .0166838443700077
 .0119814480803917
 .00148157691316671
 .00115949497552177
 0
 0
 .00644163875289874
 .00644163875289874
 .00386498325173924
 .00251223911363051
 .00186807523834063
 0
 0
 .0035429013140943
 .0035429013140943
 .00199690801339861
 .000515331100231899
 .000322081937644937
 .000322081937644937
 .00270548827621747
 .0020613244009276
 .0020613244009276
 .00025766555011595
 .00167482607575367
 .000450914712702912
 .000450914712702912
 .000193249162586962
 0
 0
 0
 .000644163875289874
 .000128832775057975
 .000128832775057975
 .000128832775057975
 0
 0
 .000128832775057975
 .000128832775057975
 0
 0
 0
 0
 .000515331100231899
 0
 .000515331100231899
 0
 0
