# Supplementary material for: Fermentation couples Chloroflexi and sulfate-reducing bacteria to Cyanobacteria in hypersaline microbial mats
Source: Front Microbiol. 2014 Feb 26;5:61. doi: 10.3389/fmicb.2014.00061 (PMC3935151; doi:10.3389/fmicb.2014.00061)
Supplement: Supplementary file 1 [file Krona_charts_supplemental.zip › OTU table krona/GNS_MC_1200_cDNA_otutable.html]

Javascript must be enabled to view this page.

magnitude
 1.00000000000005
 .937865293626545
 .00222655162816588
 .00198302254383523
 .00166991372112441
 6.95797383801837e-05
 0
 0
 .000243529084330643
 .000208739215140551
 0
 .00191344280545505
 .00191344280545505
 0
 0
 0
 0
 .000173949345950459
 0
 0
 0
 0
 0
 0
 0
 0
 0
 0
 0
 0
 .00163512385193432
 .00038268856109101
 0
 0
 0
 0
 0
 .0145073754522683
 .00038268856109101
 .00038268856109101
 3.47898691900918e-05
 .000556637907041469
 .000452268299471194
 0
 .000347898691900918
 3.47898691900918e-05
 .00974116337322571
 .00960200389646534
 .00400083495686056
 .000139159476760367
 .000104369607570276
 0
 0
 0
 0
 0
 .000834956860562204
 0
 0
 3.47898691900918e-05
 6.95797383801837e-05
 .00201781241302533
 .00194823267464514
 .00173949345950459
 0
 0
 0
 0
 0
 .000869746729752296
 .000173949345950459
 0
 0
 0
 0
 3.47898691900918e-05
 0
 0
 .00180907319788478
 .00173949345950459
 .000243529084330643
 0
 0
 0
 .000591427776231561
 0
 0
 0
 0
 0
 .000173949345950459
 .000173949345950459
 .000139159476760367
 6.95797383801837e-05
 0
 3.47898691900918e-05
 3.47898691900918e-05
 3.47898691900918e-05
 0
 .000452268299471194
 0
 0
 .000452268299471194
 .000452268299471194
 .000173949345950459
 .0894795435569164
 .00751461174505983
 .00734066239910937
 .00427915391038129
 .000278318953520735
 .000243529084330643
 0
 .00897578625104369
 .00887141664347341
 .00567074867798497
 0
 .0729195658224328
 .0727804063456724
 .00814082939048149
 .000313108822710827
 .0486710269969387
 .0210130809908155
 .011758975786251
 .000104369607570276
 .000104369607570276
 .000104369607570276
 0
 6.95797383801837e-05
 6.95797383801837e-05
 0
 .706721402727537
 .706721402727537
 .0142290564987476
 .0123851934316727
 .0029919287503479
 0
 .000208739215140551
 6.95797383801837e-05
 .00198302254383524
 .00163512385193432
 6.95797383801837e-05
 0
 0
 0
 0
 .00055663790704147
 .000243529084330643
 .000661007514611745
 .000347898691900918
 .000695797383801837
 .000521848037851378
 .000278318953520735
 .000278318953520735
 .00146117450598386
 .00093932646813248
 .000243529084330643
 .000243529084330643
 0
 0
 .000208739215140551
 0
 .671618424714733
 .665321458391324
 .0411216253826886
 0
 0
 .517360144725856
 .386202337879209
 .0483231283050376
 0
 .00111327581408294
 0
 0
 0
 .00539242972446423
 3.47898691900918e-05
 .00243529084330643
 .00156554411355413
 .000104369607570276
 .0116546061786808
 0
 .000243529084330643
 3.47898691900918e-05
 0
 .000208739215140551
 0
 6.95797383801837e-05
 .000313108822710827
 3.47898691900918e-05
 3.47898691900918e-05
 0
 0
 .000730587252991929
 0
 0
 0
 0
 0
 .00093932646813248
 .000417478430281102
 0
 0
 3.47898691900918e-05
 0
 0
 0
 .0175688839409964
 .017429724464236
 .00250487058168661
 0
 0
 .000591427776231561
 .000591427776231561
 0
 0
 0
 0
 0
 .000765377122182021
 .000765377122182021
 .000278318953520735
 .000139159476760367
 0
 0
 .000104369607570276
 0
 .000452268299471194
 0
 0
 0
 0
 0
 .00379209574172001
 .00100890620651266
 .000487058168661286
 3.47898691900918e-05
 0
 0
 0
 0
 0
 3.47898691900918e-05
 .000208739215140551
 0
 .000139159476760367
 .00278318953520735
 .00264403005844698
 .00149596437517395
 6.95797383801837e-05
 .000139159476760367
 .000139159476760367
 .000139159476760367
 0
 0
 0
 0
 0
 0
 0
 3.47898691900918e-05
 3.47898691900918e-05
 0
 0
 0
 .000208739215140551
 .000208739215140551
 .000208739215140551
 6.95797383801837e-05
 0
 3.47898691900918e-05
 3.47898691900918e-05
 3.47898691900918e-05
 0
 0
 0
 0
 0
 0
 0
 .000104369607570276
 6.95797383801837e-05
 0
 .000452268299471194
 .000452268299471194
 .000452268299471194
 .000452268299471194
 .000452268299471194
 .000452268299471194
 .000104369607570276
 .000104369607570276
 .000104369607570276
 .00142638463679377
 .000278318953520735
 .000278318953520735
 .000208739215140551
 .00114806568327303
 .00104369607570276
 .000521848037851378
 0
 0
 3.47898691900918e-05
 0
 0
 0
 .10607431116059
 .00396604508767047
 .000313108822710827
 0
 .000278318953520735
 .000104369607570276
 3.47898691900918e-05
 3.47898691900918e-05
 3.47898691900918e-05
 0
 0
 0
 0
 0
 0
 0
 0
 0
 0
 .0017047035903145
 .000417478430281102
 3.47898691900918e-05
 0
 0
 0
 .000208739215140551
 0
 0
 .000139159476760367
 0
 0
 0
 .000139159476760367
 0
 0
 0
 0
 0
 0
 .000104369607570276
 3.47898691900918e-05
 0
 0
 0
 0
 0
 0
 .00093932646813248
 .000695797383801837
 6.95797383801837e-05
 3.47898691900918e-05
 3.47898691900918e-05
 0
 0
 3.47898691900918e-05
 0
 6.95797383801837e-05
 0
 0
 0
 0
 0
 0
 0
 0
 0
 0
 0
 0
 3.47898691900918e-05
 .000104369607570276
 6.95797383801837e-05
 0
 0
 0
 0
 0
 0
 0
 0
 0
 0
 0
 0
 0
 0
 0
 0
 0
 0
 0
 6.95797383801837e-05
 6.95797383801837e-05
 0
 0
 0
 0
 0
 0
 0
 0
 0
 0
 6.95797383801837e-05
 0
 0
 0
 0
 .000417478430281102
 .000313108822710827
 0
 0
 0
 0
 0
 0
 0
 6.95797383801837e-05
 0
 0
 0
 0
 0
 0
 .000104369607570276
 0
 6.95797383801837e-05
 0
 .000208739215140551
 3.47898691900918e-05
 0
 0
 0
 0
 0
 6.95797383801837e-05
 0
 0
 0
 0
 0
 0
 0
 .00504453103256332
 .000347898691900918
 .000278318953520735
 .000173949345950459
 3.47898691900918e-05
 0
 0
 0
 0
 3.47898691900918e-05
 0
 .00400083495686056
 .00396604508767047
 .000173949345950459
 6.95797383801837e-05
 0
 .00038268856109101
 .000278318953520735
 .03785137767882
 .000104369607570276
 0
 0
 0
 0
 0
 6.95797383801837e-05
 3.47898691900918e-05
 3.47898691900918e-05
 .0180559421096577
 .0150640133593098
 .000243529084330643
 0
 .000139159476760367
 0
 0
 .000173949345950459
 3.47898691900918e-05
 0
 0
 0
 0
 0
 .000487058168661286
 3.47898691900918e-05
 .00055663790704147
 0
 0
 0
 0
 3.47898691900918e-05
 0
 0
 .00862788755914277
 .00434873364876148
 .000139159476760367
 .00222655162816588
 .000243529084330643
 3.47898691900918e-05
 3.47898691900918e-05
 0
 0
 .00789730030615084
 .00539242972446423
 .00194823267464514
 .000487058168661286
 .000208739215140551
 .000730587252991929
 0
 0
 .0592123573615368
 .0012872251600334
 .000208739215140551
 0
 0
 0
 .00038268856109101
 .000243529084330643
 3.47898691900918e-05
 3.47898691900918e-05
 3.47898691900918e-05
 0
 0
 0
 0
 0
 .000104369607570276
 0
 0
 0
 0
 0
 0
 0
 .0365293626495965
 .0298844976342889
 0
 0
 0
 0
 0
 0
 .000104369607570276
 0
 6.95797383801837e-05
 6.95797383801837e-05
 6.95797383801837e-05
 0
 0
 0
 0
 0
 0
 .00313108822710827
 0
 0
 0
 0
 0
 .00055663790704147
 3.47898691900918e-05
 0
 0
 0
 0
 .00240050097411634
 3.47898691900918e-05
 0
 6.95797383801837e-05
 6.95797383801837e-05
 3.47898691900918e-05
 .000208739215140551
 3.47898691900918e-05
 0
 0
 0
 0
 0
 0
 0
 0
 0
 0
 0
 0
 0
 0
 0
 .00281797940439744
 .00247008071249652
 .000139159476760367
 .000104369607570276
 .000417478430281102
 .000173949345950459
 0
 3.47898691900918e-05
 0
 0
 0
 0
 0
 6.95797383801837e-05
 6.95797383801837e-05
 6.95797383801837e-05
 0
 0
 0
 .00215697188978569
 .00114806568327303
 0
 0
 0
 0
 0
 .0100542721959365
 3.47898691900918e-05
 .00963679376565544
 3.47898691900918e-05
 0
 0
 0
 0
 0
 0
 .00504453103256331
 .00121764542165321
 0
 .000626217645421653
 0
 0
 .00462705260228221
 .00462705260228221
 .00393125521848038
 .00093932646813248
 6.95797383801837e-05
 0
 0
 0
 0
 .00107848594489285
 0
 0
 0
 0
 3.47898691900918e-05
 3.47898691900918e-05
 3.47898691900918e-05
 3.47898691900918e-05
 3.47898691900918e-05
 0
 0
 0
 0
 .00093932646813248
 .000487058168661286
 .000208739215140551
 .000104369607570276
 0
 0
 .000278318953520735
 .000278318953520735
 .000208739215140551
 6.95797383801837e-05
 0
 0
 .000452268299471194
 .000452268299471194
 .000208739215140551
 6.95797383801837e-05
 0
 3.47898691900918e-05
 0
 .00375730587252992
 .00375730587252992
 .00198302254383523
 .000313108822710827
 6.95797383801837e-05
 0
 .0581686612858338
 .053367659337601
 .053367659337601
 0
 .05298497077651
 .0202824937378235
 .0195866963540217
 .00789730030615085
 .000347898691900918
 .000278318953520735
 0
 .00480100194823267
 0
 0
 0
 0
 0
 0
 0
 0
 0
 .00121764542165321
 .00038268856109101
 .00278318953520735
 .000139159476760367
 .00229613136654606
 .000765377122182021
 3.47898691900918e-05
