# Supplementary material for: Fermentation couples Chloroflexi and sulfate-reducing bacteria to Cyanobacteria in hypersaline microbial mats
Source: Front Microbiol. 2014 Feb 26;5:61. doi: 10.3389/fmicb.2014.00061 (PMC3935151; doi:10.3389/fmicb.2014.00061)
Supplement: Supplementary file 1 [file Krona_charts_supplemental.zip › OTU table krona/GNS_MC_1200_DNA_otutable.html]

Javascript must be enabled to view this page.

magnitude
 .999999999999968
 .982674516400305
 .0109335576114382
 .00933557611438183
 .00925147182506308
 .00504625735912532
 .000168208578637511
 0
 .00159798149705635
 .00159798149705635
 0
 .00227081581160639
 .00227081581160639
 0
 0
 0
 0
 .000588730025231287
 0
 0
 0
 0
 0
 0
 0
 0
 0
 0
 0
 0
 .00168208578637511
 .000756938603868797
 0
 0
 0
 0
 0
 .0627417998317916
 .000168208578637511
 .000168208578637511
 0
 .000504625735912532
 .000504625735912532
 .000336417157275021
 .000168208578637511
 0
 .03919259882254
 .0391084945332212
 .0190916736753574
 .000420521446593776
 .000336417157275021
 .000168208578637511
 0
 8.41042893187553e-05
 8.41042893187553e-05
 8.41042893187553e-05
 .00100925147182506
 0
 0
 0
 8.41042893187553e-05
 .00984020185029436
 .00967199327165685
 .00647603027754416
 0
 0
 0
 0
 0
 .00168208578637511
 .000168208578637511
 0
 0
 0
 0
 .00100925147182506
 0
 0
 .0130361648444071
 .0129520605550883
 .000588730025231287
 .000252312867956266
 0
 0
 .00521446593776283
 0
 0
 0
 0
 .000420521446593776
 .00344827586206897
 .00344827586206897
 .00269133725820017
 .00193439865433137
 0
 0
 0
 0
 0
 .0024390243902439
 0
 0
 .0024390243902439
 .0024390243902439
 .00176619007569386
 .284440706476032
 .0874684608915052
 .0868797308662739
 .0507989907485283
 .00285954583683768
 .00151387720773759
 0
 .0333052985702271
 .0330529857022708
 .0229604709840202
 .000168208578637511
 .163414634146342
 .162741799831792
 .0171572750210261
 .000504625735912532
 .103784693019344
 .0518923465096721
 .0372582001682086
 0
 0
 0
 0
 .000252312867956266
 .000252312867956266
 8.41042893187553e-05
 .30756938603869
 .307485281749371
 .007905803195963
 .00605550883095038
 .00201850294365013
 0
 0
 0
 .000504625735912532
 .000336417157275021
 0
 0
 0
 0
 0
 .000252312867956266
 .000168208578637511
 .000588730025231287
 .000252312867956266
 8.41042893187553e-05
 0
 0
 0
 .000336417157275021
 .000168208578637511
 8.41042893187553e-05
 8.41042893187553e-05
 0
 0
 8.41042893187553e-05
 0
 .2784693019344
 .272161480235493
 .0317073170731707
 8.41042893187553e-05
 0
 .163919259882254
 .122624053826745
 .0147182506307822
 0
 .00159798149705635
 0
 0
 0
 .00353238015138772
 0
 .000672834314550042
 .000420521446593776
 0
 .0115222876366695
 0
 .00109335576114382
 8.41042893187553e-05
 0
 0
 0
 0
 .000672834314550042
 .000504625735912532
 0
 0
 0
 0
 0
 0
 0
 0
 0
 .000336417157275021
 8.41042893187553e-05
 0
 0
 8.41042893187553e-05
 8.41042893187553e-05
 8.41042893187553e-05
 0
 .0201009251471825
 .0200168208578637
 .00311185870479394
 0
 0
 .000252312867956266
 .000252312867956266
 8.41042893187553e-05
 8.41042893187553e-05
 8.41042893187553e-05
 8.41042893187553e-05
 8.41042893187553e-05
 .0211101766190075
 .0211101766190075
 .0207737594617325
 .0116063919259882
 .000336417157275021
 8.41042893187553e-05
 .00597140454163162
 0
 .000252312867956266
 8.41042893187553e-05
 8.41042893187553e-05
 8.41042893187553e-05
 8.41042893187553e-05
 8.41042893187553e-05
 .00513036164844407
 .00117746005046257
 .000756938603868797
 8.41042893187553e-05
 0
 0
 0
 0
 0
 0
 .000168208578637511
 0
 0
 .0039529015979815
 .0031959629941127
 .00210260723296888
 .000252312867956266
 0
 0
 0
 0
 0
 0
 0
 0
 0
 0
 0
 8.41042893187553e-05
 0
 0
 0
 .000336417157275021
 .000336417157275021
 .000336417157275021
 .000168208578637511
 0
 .000252312867956266
 .000252312867956266
 .000252312867956266
 .000168208578637511
 0
 0
 .00218671152228764
 .00218671152228764
 .00151387720773759
 8.41042893187553e-05
 0
 0
 0
 .00117746005046257
 .00117746005046257
 .00117746005046257
 .00117746005046257
 .00100925147182506
 .000925147182506308
 .000336417157275021
 .000336417157275021
 0
 .00941968040370059
 .000925147182506308
 .000925147182506308
 .000336417157275021
 .00849453322119429
 .00689655172413793
 .00193439865433137
 .000336417157275021
 .000336417157275021
 0
 0
 8.41042893187553e-05
 0
 .218082422203533
 .0197645079899074
 .000252312867956266
 8.41042893187553e-05
 .000925147182506308
 .000756938603868797
 .000252312867956266
 8.41042893187553e-05
 0
 0
 0
 0
 0
 0
 0
 0
 0
 0
 0
 .00504625735912532
 .000420521446593776
 0
 0
 0
 0
 .000588730025231287
 8.41042893187553e-05
 8.41042893187553e-05
 .00126156433978133
 0
 .000168208578637511
 0
 0
 0
 0
 .000168208578637511
 .000168208578637511
 0
 8.41042893187553e-05
 0
 0
 0
 0
 8.41042893187553e-05
 0
 0
 0
 .00681244743481918
 .00538267451640034
 8.41042893187553e-05
 8.41042893187553e-05
 .000336417157275021
 .000168208578637511
 0
 .000841042893187553
 .000168208578637511
 .000588730025231287
 0
 0
 0
 .00100925147182506
 0
 .00100925147182506
 0
 0
 0
 0
 .000252312867956266
 .000252312867956266
 0
 .000168208578637511
 0
 0
 0
 0
 0
 0
 0
 8.41042893187553e-05
 0
 0
 0
 0
 0
 0
 0
 0
 0
 0
 0
 0
 0
 0
 0
 8.41042893187553e-05
 8.41042893187553e-05
 8.41042893187553e-05
 0
 8.41042893187553e-05
 0
 0
 8.41042893187553e-05
 8.41042893187553e-05
 .000841042893187553
 .000588730025231287
 .000420521446593776
 0
 0
 .0047098402018503
 .00403700588730025
 0
 0
 0
 0
 0
 0
 0
 0
 0
 0
 0
 0
 0
 0
 0
 0
 0
 0
 .00185029436501262
 .000168208578637511
 0
 0
 0
 0
 0
 .00109335576114382
 0
 0
 0
 8.41042893187553e-05
 8.41042893187553e-05
 0
 0
 .00841042893187553
 .000420521446593776
 .000420521446593776
 0
 .000672834314550042
 0
 0
 0
 8.41042893187553e-05
 0
 0
 .00723296888141295
 .00681244743481918
 0
 0
 0
 8.41042893187553e-05
 0
 .0857863751051304
 .00126156433978133
 .000756938603868797
 .000672834314550042
 .000588730025231287
 8.41042893187553e-05
 0
 .000420521446593776
 .000252312867956266
 0
 .0560134566862911
 .0486122792262406
 .00100925147182506
 8.41042893187553e-05
 .000841042893187553
 0
 0
 .000420521446593776
 8.41042893187553e-05
 .000504625735912532
 0
 .000504625735912532
 0
 .000168208578637511
 .000588730025231287
 0
 .000925147182506308
 0
 0
 0
 .000252312867956266
 0
 0
 0
 .00857863751051303
 .00437342304457527
 .000168208578637511
 .00403700588730025
 .00117746005046257
 .000252312867956266
 8.41042893187553e-05
 .000168208578637511
 0
 .0132043734230446
 .00815811606391926
 .00260723296888141
 .00109335576114382
 .000168208578637511
 .00134566862910008
 0
 0
 .104121110176619
 .00109335576114382
 .000420521446593776
 0
 .000168208578637511
 8.41042893187553e-05
 .00344827586206897
 .00252312867956266
 .00185029436501262
 .00142977291841884
 8.41042893187553e-05
 0
 0
 0
 0
 0
 0
 0
 0
 0
 0
 0
 0
 0
 .0619007569386039
 .043650126156434
 8.41042893187553e-05
 8.41042893187553e-05
 0
 0
 0
 0
 .000504625735912532
 0
 8.41042893187553e-05
 8.41042893187553e-05
 8.41042893187553e-05
 0
 0
 0
 0
 8.41042893187553e-05
 0
 .011354079058032
 8.41042893187553e-05
 0
 0
 0
 0
 .000672834314550042
 .000672834314550042
 0
 0
 0
 0
 .00412111017661901
 0
 0
 .000168208578637511
 .000168208578637511
 8.41042893187553e-05
 .000420521446593776
 .000252312867956266
 0
 0
 0
 0
 0
 0
 0
 0
 .000336417157275021
 8.41042893187553e-05
 0
 0
 .000252312867956266
 0
 .000252312867956266
 .0063919259882254
 .00605550883095038
 .000252312867956266
 .000168208578637511
 .000336417157275021
 .000252312867956266
 0
 8.41042893187553e-05
 0
 0
 0
 0
 0
 0
 0
 0
 0
 0
 0
 .00521446593776283
 .00218671152228764
 8.41042893187553e-05
 0
 .000252312867956266
 .000252312867956266
 0
 .0175777964676198
 0
 .0162321278385198
 .000168208578637511
 8.41042893187553e-05
 0
 0
 0
 0
 0
 .00580319596299412
 .000925147182506308
 0
 .00117746005046257
 0
 0
 .0396131202691338
 .0396131202691338
 .039529015979815
 .0335576114381833
 .00933557611438183
 .000756938603868797
 .00151387720773759
 .000336417157275021
 .000168208578637511
 .00193439865433137
 0
 8.41042893187553e-05
 8.41042893187553e-05
 0
 0
 0
 0
 0
 0
 0
 0
 0
 0
 .00689655172413793
 .00580319596299411
 .00210260723296888
 .000756938603868797
 0
 0
 .00370058873002523
 .00311185870479394
 .00185029436501262
 .00100925147182506
 0
 0
 .00109335576114382
 .00109335576114382
 .000588730025231287
 8.41042893187553e-05
 0
 .000420521446593776
 0
 .00403700588730025
 .00403700588730025
 .00269133725820017
 .000168208578637511
 .000168208578637511
 0
 .0158957106812447
 .015811606391926
 .015811606391926
 0
 .015811606391926
 .00454163162321278
 .00445752733389403
 .00227081581160639
 0
 0
 0
 8.41042893187553e-05
 0
 0
 0
 0
 0
 0
 0
 0
 0
 0
 0
 8.41042893187553e-05
 0
 8.41042893187553e-05
 0
 0
