# Supplementary material for: Fermentation couples Chloroflexi and sulfate-reducing bacteria to Cyanobacteria in hypersaline microbial mats
Source: Front Microbiol. 2014 Feb 26;5:61. doi: 10.3389/fmicb.2014.00061 (PMC3935151; doi:10.3389/fmicb.2014.00061)
Supplement: Supplementary file 1 [file Krona_charts_supplemental.zip › OTU table krona/GNS_MC_2400_cDNA_otutable.html]

Javascript must be enabled to view this page.

magnitude
 1
 .985266872518749
 .000838112042346714
 .000838112042346714
 .000749889722099691
 .000308778120864579
 0
 0
 0
 0
 0
 .000176444640494045
 .000176444640494045
 0
 0
 0
 0
 0
 0
 0
 0
 0
 0
 0
 0
 0
 0
 0
 0
 0
 .000176444640494045
 0
 0
 0
 0
 0
 0
 .011645346272607
 .000441111601235112
 .000441111601235112
 0
 .000176444640494045
 .000176444640494045
 0
 .000132333480370534
 0
 .00917512130569033
 .00917512130569033
 .00348478164975739
 0
 0
 0
 0
 0
 0
 0
 .000926334362593736
 0
 0
 0
 0
 .000661667401852669
 .000661667401852669
 .000573445081605646
 0
 0
 0
 0
 0
 .000308778120864579
 0
 0
 0
 0
 0
 4.41111601235113e-05
 0
 0
 .0011910013233348
 .0011910013233348
 .000176444640494045
 0
 0
 0
 8.82223202470225e-05
 0
 8.82223202470225e-05
 4.41111601235113e-05
 4.41111601235113e-05
 0
 4.41111601235113e-05
 4.41111601235113e-05
 4.41111601235113e-05
 4.41111601235113e-05
 0
 0
 0
 0
 0
 .000132333480370534
 0
 0
 .000132333480370534
 .000132333480370534
 8.82223202470225e-05
 .0256726951918835
 .00388178209086899
 .00383767093074548
 .00211733568592854
 .000220555800617556
 .000220555800617556
 0
 .00269078076753419
 .00264666960741068
 .00189677988531098
 0
 .0190560211733568
 .0189236876929863
 .00176444640494045
 0
 .0129245699161888
 .00586678429642699
 .00388178209086899
 8.82223202470225e-05
 8.82223202470225e-05
 8.82223202470225e-05
 0
 4.41111601235113e-05
 4.41111601235113e-05
 0
 .873400970445526
 .873400970445526
 .015527128363476
 .0137626819585355
 .00423467137185708
 0
 .000220555800617556
 4.41111601235113e-05
 .00299955888839876
 .000794000882223202
 .000132333480370534
 0
 0
 4.41111601235113e-05
 4.41111601235113e-05
 .00141155712395236
 .000794000882223202
 .000132333480370534
 0
 .000176444640494045
 .000176444640494045
 .000176444640494045
 .000176444640494045
 .00141155712395236
 .00105866784296427
 .00035288928098809
 .00035288928098809
 0
 0
 0
 8.82223202470225e-05
 .823599470666082
 .82055580061756
 .0116894574327305
 0
 0
 .706837229819147
 .568107631230703
 .0489633877370975
 0
 .00176444640494045
 0
 0
 0
 .00749889722099691
 0
 .00132333480370534
 .000882223202470225
 .000132333480370534
 .0209528010586678
 0
 .00114689016321129
 4.41111601235113e-05
 0
 0
 0
 0
 .00035288928098809
 8.82223202470225e-05
 .000617556241729157
 0
 0
 .000264666960741068
 0
 0
 0
 0
 0
 .000308778120864579
 .000176444640494045
 0
 4.41111601235113e-05
 4.41111601235113e-05
 4.41111601235113e-05
 0
 0
 .0323334803705337
 .0322452580502867
 .00383767093074548
 0
 0
 .000882223202470225
 .000882223202470225
 0
 0
 0
 0
 0
 .000264666960741067
 .000264666960741067
 4.41111601235113e-05
 4.41111601235113e-05
 0
 0
 0
 0
 .000176444640494045
 0
 0
 0
 0
 0
 .00198500220555801
 .000926334362593736
 .000264666960741067
 4.41111601235113e-05
 0
 0
 0
 0
 0
 0
 .000220555800617556
 .000176444640494045
 4.41111601235113e-05
 .00105866784296427
 .000970445522717247
 .000661667401852669
 0
 0
 0
 0
 0
 0
 0
 0
 0
 0
 0
 0
 4.41111601235113e-05
 0
 0
 0
 0
 0
 0
 0
 0
 0
 0
 0
 0
 0
 0
 4.41111601235113e-05
 4.41111601235113e-05
 4.41111601235113e-05
 0
 0
 0
 0
 0
 0
 0
 0
 0
 0
 0
 0
 0
 .000617556241729157
 .000132333480370534
 .000132333480370534
 8.82223202470225e-05
 .000485222761358624
 .000485222761358624
 8.82223202470225e-05
 0
 0
 0
 0
 0
 0
 .0647110719011909
 .00185266872518747
 8.82223202470225e-05
 0
 .000308778120864579
 .000220555800617556
 .000132333480370534
 8.82223202470225e-05
 8.82223202470225e-05
 0
 0
 0
 0
 0
 0
 0
 0
 0
 0
 .000308778120864579
 4.41111601235113e-05
 4.41111601235113e-05
 0
 0
 0
 0
 0
 0
 0
 0
 0
 0
 0
 0
 0
 0
 0
 0
 0
 0
 0
 0
 0
 0
 0
 0
 0
 .000441111601235112
 .00035288928098809
 0
 0
 8.82223202470225e-05
 0
 0
 0
 0
 0
 0
 0
 0
 4.41111601235113e-05
 0
 4.41111601235113e-05
 0
 0
 0
 0
 0
 0
 0
 0
 0
 0
 0
 0
 0
 0
 0
 0
 0
 0
 0
 0
 0
 0
 0
 0
 0
 0
 0
 0
 0
 0
 0
 0
 0
 0
 0
 0
 0
 0
 0
 0
 8.82223202470225e-05
 0
 0
 0
 0
 .000485222761358624
 .000441111601235112
 0
 0
 0
 0
 0
 0
 0
 4.41111601235113e-05
 0
 0
 0
 0
 0
 0
 0
 0
 0
 0
 .000220555800617556
 0
 0
 0
 0
 0
 0
 .000176444640494045
 0
 0
 0
 0
 0
 0
 0
 .00374944861049846
 .000661667401852669
 .000661667401852669
 4.41111601235113e-05
 4.41111601235113e-05
 0
 0
 0
 0
 0
 0
 .00299955888839877
 .00299955888839877
 0
 0
 0
 4.41111601235113e-05
 0
 .0200264666960741
 .000132333480370534
 4.41111601235113e-05
 0
 0
 0
 0
 8.82223202470225e-05
 8.82223202470225e-05
 4.41111601235113e-05
 .0109395677106308
 .00939567710630789
 0
 0
 4.41111601235113e-05
 0
 0
 .000220555800617556
 4.41111601235113e-05
 4.41111601235113e-05
 0
 4.41111601235113e-05
 4.41111601235113e-05
 0
 .000485222761358624
 0
 8.82223202470225e-05
 0
 0
 0
 0
 0
 0
 0
 .00405822673136304
 .00172033524481694
 0
 .000882223202470225
 .000529333921482135
 4.41111601235113e-05
 0
 0
 0
 .00366122629025143
 .00264666960741068
 .000661667401852669
 8.82223202470225e-05
 4.41111601235113e-05
 4.41111601235113e-05
 8.82223202470225e-05
 8.82223202470225e-05
 .0389942655491839
 .0011910013233348
 .00035288928098809
 0
 0
 0
 .000264666960741068
 .000176444640494045
 4.41111601235113e-05
 4.41111601235113e-05
 0
 8.82223202470225e-05
 0
 0
 0
 0
 0
 0
 0
 0
 0
 0
 0
 0
 .0245258050286722
 .0205999117776797
 .000132333480370534
 .000132333480370534
 4.41111601235113e-05
 0
 0
 0
 0
 0
 4.41111601235113e-05
 8.82223202470225e-05
 8.82223202470225e-05
 0
 0
 0
 0
 0
 0
 .00189677988531098
 0
 0
 0
 0
 0
 4.41111601235113e-05
 4.41111601235113e-05
 0
 0
 0
 0
 .00189677988531098
 0
 0
 .000220555800617556
 .000176444640494045
 0
 0
 0
 0
 0
 0
 0
 0
 0
 0
 0
 0
 0
 0
 0
 0
 0
 0
 .00176444640494045
 .00176444640494045
 0
 0
 4.41111601235113e-05
 0
 0
 0
 0
 0
 0
 0
 0
 4.41111601235113e-05
 4.41111601235113e-05
 4.41111601235113e-05
 0
 0
 0
 .00141155712395236
 .000838112042346714
 0
 0
 0
 0
 0
 .00732245258050287
 8.82223202470225e-05
 .00701367445963829
 .000264666960741067
 0
 0
 0
 0
 0
 0
 .00224966916629907
 .000749889722099691
 0
 .000264666960741067
 0
 0
 .00326422584913983
 .00326422584913983
 .00291133656815174
 .000661667401852669
 8.82223202470225e-05
 0
 0
 4.41111601235113e-05
 0
 .000749889722099691
 .000176444640494045
 0
 0
 0
 0
 0
 0
 0
 0
 0
 0
 0
 0
 .000485222761358624
 .000397000441111601
 8.82223202470225e-05
 8.82223202470225e-05
 0
 0
 .000308778120864579
 .000308778120864579
 .000176444640494045
 4.41111601235113e-05
 0
 0
 8.82223202470225e-05
 8.82223202470225e-05
 8.82223202470225e-05
 0
 0
 4.41111601235113e-05
 0
 .00198500220555801
 .00198500220555801
 .000794000882223202
 4.41111601235113e-05
 4.41111601235113e-05
 0
 .0141596823996471
 .013806793118659
 .013806793118659
 0
 .013806793118659
 .00882223202470225
 .0086457873842082
 .000882223202470225
 0
 0
 0
 .00035288928098809
 0
 0
 0
 0
 0
 0
 0
 0
 0
 0
 0
 .00035288928098809
 0
 .00035288928098809
 0
 0
