# Supplementary material for: Fermentation couples Chloroflexi and sulfate-reducing bacteria to Cyanobacteria in hypersaline microbial mats
Source: Front Microbiol. 2014 Feb 26;5:61. doi: 10.3389/fmicb.2014.00061 (PMC3935151; doi:10.3389/fmicb.2014.00061)
Supplement: Supplementary file 1 [file Krona_charts_supplemental.zip › OTU table krona/GNS_MC_2400_DNA_otutable.html]

Javascript must be enabled to view this page.

magnitude
 .999999999999952
 .976133324188966
 .00630958096152527
 .0050750977299225
 .0050750977299225
 .00130306563335848
 6.85824017557095e-05
 6.85824017557095e-05
 .00123448323160277
 .00123448323160277
 0
 .00267471366847267
 .00267471366847267
 0
 0
 0
 0
 .000548659214045676
 .000137164803511419
 .000137164803511419
 0
 0
 0
 .000137164803511419
 0
 0
 0
 6.85824017557095e-05
 0
 0
 .00198888965091557
 .000137164803511419
 0
 0
 6.85824017557095e-05
 6.85824017557095e-05
 6.85824017557095e-05
 .0572663054660175
 .000548659214045676
 .000548659214045676
 0
 .000137164803511419
 .000137164803511419
 6.85824017557095e-05
 0
 0
 .0342912008778548
 .0342226184760991
 .012756326726562
 .000548659214045676
 .000480076812289966
 0
 0
 0
 0
 0
 .0028804608737398
 0
 0
 0
 0
 .00932720663877649
 .00925862423702078
 .00500651532816679
 0
 0
 0
 0
 0
 .000480076812289966
 0
 6.85824017557095e-05
 6.85824017557095e-05
 6.85824017557095e-05
 0
 .000205747205267128
 0
 0
 .0129620739318291
 .0128934915300734
 .0021946368561827
 6.85824017557095e-05
 0
 0
 .00322337288251835
 0
 .000205747205267128
 .000137164803511419
 6.85824017557095e-05
 .000480076812289966
 .00281187847198409
 .00281187847198409
 .00205747205267128
 .000137164803511419
 0
 6.85824017557095e-05
 6.85824017557095e-05
 6.85824017557095e-05
 6.85824017557095e-05
 .00137164803511419
 6.85824017557095e-05
 6.85824017557095e-05
 .00130306563335848
 .00130306563335848
 .000960153624579933
 .310472532748096
 .10582264590906
 .104725327480969
 .0646732048556341
 .00418352650709828
 .0021946368561827
 6.85824017557095e-05
 .04156093546396
 .04060078183938
 .0150195459845004
 0
 .162334544955761
 .161442973732937
 .0385433097867087
 .00411494410534257
 .0730402578698307
 .0425210890885399
 .026678554282971
 .000205747205267128
 .000205747205267128
 .000137164803511419
 0
 .000685824017557095
 .000685824017557095
 .000480076812289966
 .222481311295517
 .222481311295517
 .00809272340717372
 .00582950414923531
 .000685824017557095
 0
 .00123448323160277
 0
 .000205747205267128
 .000137164803511419
 .000137164803511419
 0
 0
 0
 0
 .000342912008778547
 .000205747205267128
 .000274329607022838
 .000137164803511419
 6.85824017557095e-05
 6.85824017557095e-05
 0
 0
 .000617241615801385
 .000137164803511419
 0
 0
 0
 0
 6.85824017557095e-05
 0
 .186818462382548
 .175845278101635
 .0132364035388519
 0
 6.85824017557095e-05
 .0818188052945615
 .0633701392222756
 .00294904327549551
 6.85824017557095e-05
 .0139908099581647
 .00308620807900693
 .00226321925793841
 0
 .000274329607022838
 0
 .000342912008778547
 .000274329607022838
 0
 .00212605445442699
 .000137164803511419
 .000342912008778547
 0
 0
 .00116590082984706
 0
 0
 .000137164803511419
 .000137164803511419
 6.85824017557095e-05
 .000205747205267128
 .000205747205267128
 0
 0
 0
 0
 0
 0
 .000891571222824223
 .000754406419312804
 0
 0
 .000137164803511419
 6.85824017557095e-05
 0
 0
 .0256498182566354
 .0209862149372471
 .00994444825457788
 0
 0
 0
 0
 0
 0
 0
 0
 0
 .0162540292161032
 .0162540292161032
 .016048282010836
 .00884712982648653
 0
 0
 .00384061449831973
 6.85824017557095e-05
 .000205747205267128
 0
 0
 0
 0
 0
 .00404636170358686
 .00178314244564845
 .00116590082984706
 0
 0
 0
 0
 0
 0
 0
 .000274329607022838
 0
 0
 .00226321925793841
 .00185172484740416
 .00102873602633564
 0
 .000274329607022838
 .000274329607022838
 .000274329607022838
 0
 0
 0
 0
 0
 0
 0
 0
 .000137164803511419
 0
 0
 0
 .000411494410534257
 .000411494410534257
 .000411494410534257
 .000137164803511419
 0
 .000548659214045676
 .000548659214045676
 .000548659214045676
 .000480076812289966
 0
 0
 .00137164803511419
 .00137164803511419
 .000960153624579933
 0
 6.85824017557095e-05
 6.85824017557095e-05
 6.85824017557095e-05
 .00130306563335848
 .00130306563335848
 .00130306563335848
 .00123448323160277
 .000754406419312804
 .000754406419312804
 .000480076812289966
 .000480076812289966
 .000137164803511419
 .0126191619230506
 .00308620807900693
 .00308620807900693
 .00109731842809135
 .00953295384404363
 .00857280021946369
 .00377203209656402
 0
 0
 6.85824017557095e-05
 6.85824017557095e-05
 0
 0
 .272409299773675
 .0329881352444963
 .000342912008778547
 .000137164803511419
 .00178314244564845
 .000342912008778547
 .000205747205267128
 0
 0
 6.85824017557095e-05
 6.85824017557095e-05
 0
 0
 0
 0
 0
 0
 0
 0
 .00637816336328099
 .00178314244564845
 .000411494410534257
 0
 0
 0
 .000548659214045676
 6.85824017557095e-05
 6.85824017557095e-05
 .000685824017557095
 0
 6.85824017557095e-05
 0
 .000480076812289966
 6.85824017557095e-05
 .000205747205267128
 0
 0
 0
 0
 6.85824017557095e-05
 0
 0
 0
 6.85824017557095e-05
 6.85824017557095e-05
 6.85824017557095e-05
 6.85824017557095e-05
 .0141965571634319
 .0115904258967149
 .000891571222824223
 .000205747205267128
 .000548659214045676
 0
 0
 .00102873602633564
 6.85824017557095e-05
 .000480076812289966
 0
 .000205747205267128
 .000205747205267128
 .000274329607022838
 6.85824017557095e-05
 6.85824017557095e-05
 0
 6.85824017557095e-05
 0
 6.85824017557095e-05
 .00171456004389274
 .000411494410534257
 0
 .000137164803511419
 0
 6.85824017557095e-05
 .000137164803511419
 .000137164803511419
 0
 0
 0
 .000137164803511419
 0
 0
 0
 0
 6.85824017557095e-05
 0
 0
 0
 6.85824017557095e-05
 0
 0
 0
 0
 0
 6.85824017557095e-05
 0
 6.85824017557095e-05
 6.85824017557095e-05
 0
 0
 0
 0
 6.85824017557095e-05
 6.85824017557095e-05
 .000342912008778547
 6.85824017557095e-05
 6.85824017557095e-05
 0
 0
 .00816130580892943
 .00706398738083808
 .0021946368561827
 .00198888965091558
 .000205747205267128
 0
 0
 0
 0
 0
 6.85824017557095e-05
 6.85824017557095e-05
 0
 0
 0
 0
 6.85824017557095e-05
 0
 6.85824017557095e-05
 0
 .00198888965091557
 .00109731842809135
 .000685824017557095
 6.85824017557095e-05
 .000480076812289966
 0
 0
 .000685824017557095
 0
 0
 6.85824017557095e-05
 0
 0
 .000137164803511419
 0
 .0114532610932035
 .000342912008778547
 .000342912008778547
 0
 .00185172484740416
 0
 0
 0
 .000205747205267128
 .000342912008778547
 .000342912008778547
 .00870996502297511
 .00164597764213703
 6.85824017557095e-05
 6.85824017557095e-05
 0
 6.85824017557095e-05
 0
 .0888827926753996
 .00116590082984706
 .000685824017557095
 .000342912008778547
 .000205747205267128
 .000205747205267128
 0
 .000274329607022838
 0
 6.85824017557095e-05
 .0545915917975448
 .0417666826692271
 .00116590082984706
 .000754406419312804
 .00109731842809135
 0
 0
 6.85824017557095e-05
 .000205747205267128
 .000685824017557095
 .000137164803511419
 .000480076812289966
 6.85824017557095e-05
 .000205747205267128
 .000205747205267128
 0
 .00205747205267128
 6.85824017557095e-05
 6.85824017557095e-05
 0
 .000137164803511419
 .000205747205267128
 .000137164803511419
 .000137164803511419
 .0168026884301488
 .00699540497908237
 .000411494410534257
 .00329195528427406
 .00178314244564845
 .0014402304368699
 .000137164803511419
 0
 0
 .00960153624579933
 .00480076812289967
 .00157739524038132
 0
 0
 .00102873602633564
 6.85824017557095e-05
 6.85824017557095e-05
 .139016528358822
 .00157739524038132
 .000342912008778547
 6.85824017557095e-05
 .000137164803511419
 6.85824017557095e-05
 .011796173101982
 .00905287703175366
 .00123448323160277
 .000617241615801385
 .000205747205267128
 .000205747205267128
 .000205747205267128
 6.85824017557095e-05
 6.85824017557095e-05
 0
 0
 .000822988821068514
 0
 .000274329607022838
 .000274329607022838
 6.85824017557095e-05
 0
 0
 .0786640148137988
 .0443728139359441
 .00171456004389274
 .000960153624579933
 6.85824017557095e-05
 6.85824017557095e-05
 .000342912008778547
 0
 6.85824017557095e-05
 0
 6.85824017557095e-05
 .000822988821068514
 .000342912008778547
 0
 6.85824017557095e-05
 0
 0
 6.85824017557095e-05
 0
 .0247582470338111
 .000617241615801385
 0
 .000274329607022838
 6.85824017557095e-05
 0
 .000891571222824223
 0
 0
 0
 0
 0
 .000891571222824224
 6.85824017557095e-05
 0
 .000137164803511419
 6.85824017557095e-05
 6.85824017557095e-05
 .00178314244564845
 .00109731842809135
 0
 0
 0
 0
 0
 0
 .000274329607022838
 6.85824017557095e-05
 .000411494410534257
 .000274329607022838
 0
 0
 .000137164803511419
 0
 6.85824017557095e-05
 .00809272340717372
 .00301762567725122
 .000205747205267128
 .000205747205267128
 .00130306563335848
 .000685824017557095
 .000205747205267128
 0
 6.85824017557095e-05
 .000137164803511419
 0
 0
 0
 0
 0
 0
 6.85824017557095e-05
 .000205747205267128
 0
 .00617241615801386
 .00164597764213703
 .000137164803511419
 .000137164803511419
 0
 0
 0
 .0227693573828956
 .00747548179137233
 .0115218434949592
 6.85824017557095e-05
 0
 .00109731842809135
 .000822988821068514
 .000822988821068514
 0
 0
 .00452643851587683
 .00150881283862561
 0
 .000274329607022838
 0
 0
 .0473904396131953
 .0473904396131953
 .0473904396131953
 .0343597832796105
 .0103559426651121
 .000342912008778547
 .000411494410534257
 .000274329607022838
 .000205747205267128
 .00123448323160277
 .000137164803511419
 .000205747205267128
 .000205747205267128
 6.85824017557095e-05
 0
 0
 0
 0
 0
 0
 0
 0
 0
 .0129620739318291
 .0108360194774021
 .00301762567725122
 .000960153624579933
 0
 0
 .00781839380015089
 .00486935052465538
 .000480076812289966
 .000411494410534257
 .000137164803511419
 .000137164803511419
 .00198888965091558
 .00198888965091558
 .00157739524038132
 .000822988821068514
 .000548659214045676
 0
 0
 .00253754886496125
 .00253754886496125
 .00171456004389274
 6.85824017557095e-05
 0
 0
 .0215348741512928
 .0214662917495371
 .0214662917495371
 0
 .0214662917495371
 .00706398738083808
 .00678965777381524
 .00137164803511419
 0
 0
 0
 6.85824017557095e-05
 0
 0
 0
 0
 0
 0
 0
 0
 0
 0
 0
 6.85824017557095e-05
 0
 6.85824017557095e-05
 0
 0
